# Alpha-synuclein suppresses mitochondrial protease ClpP to trigger mitochondrial oxidative damage and neurotoxicity

**DOI:** 10.1007/s00401-019-01993-2

**Published:** 2019-03-15

**Authors:** Di Hu, Xiaoyan Sun, Xudong Liao, Xinwen Zhang, Sara Zarabi, Aaron Schimmer, Yuning Hong, Christopher Ford, Yu Luo, Xin Qi

**Affiliations:** 1 Department of Physiology and Biophysics, Case Western Reserve University School of Medicine, 10900 Euclid Ave, E516, Cleveland, OH 44106-4970 USA; 2 Case Cardiovascular Research Institute, Case Western Reserve University School of Medicine, Cleveland, USA; 3 Center of Implant Dentistry, School of Stomatology, China Medical University, Shenyang, 110002 China; 4 Princess Margaret Cancer Centre, Toronto, ON M5G 2M9 Canada; 5 Department of Chemistry and Physics, La Trobe Institute for Molecular Science, La Trobe University, Melbourne, VIC 3083 Australia; 6 Department of Pharmacology, University of Colorado, Denver, CO USA; 7 Department of Molecular Genetics, University of Cincinnati, Cincinnati, OH USA; 8 Harrington Heart and Vascular Institute, University Hospitals Cleveland Medical Center, Cleveland, USA

**Keywords:** Mitochondria, Alpha-synuclein, ATP-dependent Clp protease, Parkinson’s disease

## Abstract

Both α-Synuclein (αSyn) accumulation and mitochondrial dysfunction have been implicated in the pathology of Parkinson’s disease (PD). Although studies suggest that αSyn and its missense mutant, A53T, preferentially accumulate in the mitochondria, the mechanisms by which αSyn and mitochondrial proteins regulate each other to trigger mitochondrial and neuronal toxicity are poorly understood. ATP-dependent Clp protease (ClpP), a mitochondrial matrix protease, plays an important role in regulating mitochondrial protein turnover and bioenergetics activity. Here, we show that the protein level of ClpP is selectively decreased in αSyn-expressing cell culture and neurons derived from iPS cells of PD patient carrying αSyn A53T mutant, and in dopaminergic (DA) neurons of αSyn A53T mice and PD patient postmortem brains. Deficiency in ClpP induces an overload of mitochondrial misfolded/unfolded proteins, suppresses mitochondrial respiratory activity, increases mitochondrial oxidative damage and causes cell death. Overexpression of ClpP reduces αSyn-induced mitochondrial oxidative stress through enhancing the level of Superoxide Dismutase-2 (SOD2), and suppresses the accumulation of αSyn S129 phosphorylation and promotes neuronal morphology in neurons derived from PD patient iPS cells carrying αSyn A53T mutant. Moreover, we find that αSyn WT and A53T mutant interact with ClpP and suppress its peptidase activity. The binding of αSyn to ClpP further promotes a distribution of ClpP from soluble to insoluble cellular fraction in vitro and in vivo, leading to reduced solubility of ClpP. Compensating for the loss of ClpP in the substantia nigra of αSyn A53T mice by viral expression of ClpP suppresses mitochondrial oxidative damage, and reduces αSyn pathology and behavioral deficits of mice. Our findings provide novel insights into the mechanism underlying αSyn-induced neuronal pathology, and they suggest that ClpP might be a useful therapeutic target for PD and other synucleinopathies.

**Electronic supplementary material:**

The online version of this article (10.1007/s00401-019-01993-2) contains supplementary material, which is available to authorized users.

## Introduction

Parkinson’s disease (PD), one of the most common neurodegenerative disorders, is characterized by progressive degeneration of nigrostriatal dopaminergic (DA) neurons in the midbrain. Although the mechanisms underlying PD remain elusive, α-Synuclein (αSyn) accumulation and mitochondrial dysfunction have been recognized as major contributors [1, 57]. Numerous studies suggest a key role for αSyn in the pathogenesis of PD. Missense point mutations, such as A53T and A30P, have been identified in families with autosomal dominant PD. Moreover, αSyn accumulates in the Lewy bodies and dystrophic neurites of all patients with idiopathic PD [63, 88], implicating the protein in sporadic as well as familial forms of the disease.

Substantial evidence has shown that αSyn toxicity may directly disrupt mitochondrial function. αSyn contains a cryptic mitochondrial targeting sequence [23] and is enriched in mitochondria in the striatum and substantia nigra (SN), the vulnerable brain regions in PD, compared to mitochondria in other brain regions [23, 50, 90]. FRET (fluorescence resonance energy transfer) imaging analysis further demonstrates that αSyn preferentially binds to mitochondria over synaptic membranes [59]. In mitochondria, αSyn is widely distributed in the matrix, mitochondrial inner membrane (IMM), outer membrane (OMM), and mitochondria-associated ER membrane (MAM) [23, 32, 50, 90]. αSyn wildtype (WT) and its missense mutant, A53T, have been found to reduce the activity of complex I of the electron transport chain (ETC) [17, 23], dysregulate Ca^2+^ homeostasis [12, 61, 70], promote mitochondrial fragmentation [32, 58], and increase oxidative stress [66, 78] in both cell culture and animals. Oligomeric and post-translationally modified species of αSyn can impair mitochondrial protein import [24]. Further, mice with αSyn A53T mutation show increased mitochondrial DNA damage [53] and mitophagy [14, 17, 74]. In addition, αSyn may induce mitochondrial dysfunction via decreased levels of the mitochondrial biogenesis factor PGC-1α [72]. Despite these findings suggesting that mitochondria might be a key link between αSyn toxicity and neuronal degeneration in PD, the field still lacks an understanding of how αSyn abnormality and mitochondrial functional deficiency influence each other.

Mitochondrial protein homeostasis is maintained by a group of proteases and chaperones [4, 86]. Mitochondrial matrix proteases regulate essential functions, including the ETC activity, mitochondrial protein translation, mitochondrial genome stability, and metabolism [30, 67]. Two mitochondrial proteases that regulate proteostasis in the matrix are the ATP-dependent Clp protease (ClpP) and Lon protease (LonP). LonP has been reported to stabilize mitochondrial nucleoids and to remove oxidized mitochondrial proteins in the matrix [30]. ClpP forms complexes with ClpX, an AAA^+^ chaperone, to function as an active protease ClpXP [5, 45]. ClpXP is a highly conserved proteasome-like machinery present in all bacterial species and in the mitochondria of eukaryotic cells [5]. In *C. elegans*, ClpP participates in the mitochondrial unfolded protein response (UPR^mt^), a retrograde signaling pathway that governs mitochondrial proteostasis in response to stress [37, 38, 40]. Recent proteomic analyses of mammalian cells also show that ClpP is required for the turnover of mitochondrial inner membrane and matrix proteins, most of which are components of mitochondrial ETC, subunits of the mitochondrial ribosome active in translation or enzymes involved in mitochondrial metabolism [26, 83]. Worms and mammalian cells lacking ClpP are sensitive to mitochondrial perturbations and exhibit bioenergetic defects [21, 38]. Mice deficient in ClpP exhibit mitochondrial dysfunction, diminished spontaneous motor activity, a strong inflammatory response, and decreased survival [29]. Further, ClpP mRNA and protein levels are down-regulated in response to an HSP60 mutation that results in spastic paraplegia [11, 36]. A progressive increase of ClpP protein level has been associated with frataxin deficiency in Friedreich’s ataxia [34]. These lines of evidence collectively suggest an important role of ClpP in both maintaining mitochondrial quality and neurodegenerative diseases [28, 54].

αSyn is a 14 kDa protein prone to form unfolded toxic species [6]. Because ClpP is a key regulator of mitochondrial proteostasis and αSyn is accumulated in the mitochondria [23, 26], we investigate whether αSyn affects ClpP, with which to influence mitochondrial function and neuronal survival, using in vitro and in vivo models of αSyn-associated PD. Here, we report that the protein but not the mRNA level of ClpP selectively decreases in αSyn-associated PD models in vitro and in vivo, as well as in PD patient postmortem brains. We also demonstrate a direct interaction between αSyn and ClpP, which impairs ClpP peptidase activity and promotes a distribution of ClpP from soluble to insoluble fraction. Notably, we show that compensating for the loss of ClpP in the neurons derived from iPS cells of PD patient carrying αSyn A53T mutant and the SN of αSyn A53T mice reduces αSyn-associated pathology.

## Materials and methods

### Antibodies and reagents

Protein phosphatase inhibitor and protease inhibitor cocktails were purchased from Sigma–Aldrich. Antibodies for ClpX (ab168338, 1:2000), ClpP (ab124822, 1:1000), VDAC (ab34726, 1:1000), α-Synuclein (ab27766, 1:1000), α-Synuclein (ab138501, 1:3000) and α-Synuclein phosphor S129 (ab168381, 1:1000) were from Abcam. GFP (sc-9996, 1:1000), c-Myc (sc-40, 1:1000), α-Synuclein (sc-12767, 1:1000), Enolase (sc-15343, 1:1000) and HSP60 (sc-13115, 1:2000) were from Santa Cruz Biotechnology. β-Actin (A1978, 1:10000) was from Sigma–Aldrich. TH (MAB318, 1:1000) was from Millipore. ClpP (GTX115070, 1:200) was from Genetex. ClpP (NBP1-89557, 1:200) was from Novus. LonP (15440-1-AP, 1:2000), ERAL1 (11478-1-AP, 1:2000), CHCHD3 (25624-1-AP, 1:1000) and WFS1 (11558-1-AP, 1:1000) were from Proteintech. SOD2 (611580, 1:1000) was from BD bioscience. MFN1 (H00055669-M04, 1:1000) was from Abnova. Sirt3 (5490S, 1:1000) was from cell signaling technology.

### Cell culture

SH-SY5Y cells stably expressing GFP, GFP-αSyn WT or GFP-αSyn A53T were cultured in DMEM/F12 (1:1) supplemented with 10% FBS, 100 µg/ml penicillin, 100 µg/ml streptomycin, and 400 µg/ml G418. Cells were grown at 37 °C in a 5% CO_2_ incubator. HEK293 and HeLa cells were maintained in DMEM supplemented with 10% FBS and 1% (v/v) penicillin/streptomycin.

### Induced pluripotent stem cells and neuronal differentiation

PD iPS cells lines (αSyn A53T, NN0004337) and its isogenic corrected control line (NN0004344) were obtained from RUCDR Infinite Biologics. The iPS cells were differentiated into dopaminergic neuron-enriched neuronal culture with the protocol described previously [60, 80]. Briefly, iPS cell colonies were disassociated with accutase (Invitrogen), plated onto six-well plates pre-coated with 2.5% Matrigel (BD Biosciences) and allowed to reach 80% confluence in mTeSR medium (Stem Cell Technology). For the first 7 days, cells were treated with SB431542 (10 uM; Tocris Bioscience) and Noggin (100 ng/ml) in Neural Media (NM) with FGF2 (20 ng/μl) and EGF (20 ng/μl). NM media contained: Neurobasal and DMEM/F12 (1:1), B-27 Supplement Minus Vitamin A (50×, Invitrogen), N2 Supplement (100×, Invitrogen), GlutaMAX (Invitrogen, 100×), 100 units/ml penicillin and 100 μg/ml streptomycin (Fisher); for the next 4 days, cells were treated with human recombinant Sonic hedgehog (SHH, 200 ng/ml) in neuronal differentiation medium. Neuronal differentiation medium contained Neurobasal and DMEM/F12 (1:3), B27, N2, GlutaMax and PS. In the following 3 days, cells were switched to BDNF (20 ng/ml), ascorbic acid (200 uM, Sigma–Alderich), SHH (200 ng/ml), and FGF8b (100 ng/ml) in neuronal differentiation medium. Thereafter, cells were treated with BDNF, ascorbic acid, GDNF (10 ng/ml), TGF-b (1 ng/ml), and cAMP (500 uM, Sigma–Aldrich). All growth factors were purchased from Pepro Tech (Rocky Hill, NJ, USA). Neurons were passed onto fresh plates after 20 days of induction, and at 40 days after differentiation, the cells were passed on the cover slides coated with poly-d-lysine (50 ug/ml) and Laminin (5 ug/ml), and were fixed for immunostaining or mitochondrial function assays.

For immunostaining assay, the cells were fixed and stained with TH (AB152, 1:500, Millipore) (MAB318, 1:500, Millipore), Tuj1 (MMS-435P, 1:500, Covance/801201, 1:500, Biolegend), MAP2 (4542, 1:1000, Cell Signaling), and αSyn pS129 (825701, 1:5000, Biolegend). The imaging was observed by confocal microscope (Fluoview FV100, Olympus). To analyze general neurite length, cells were stained with anti-Tuj1 (a mature neuronal marker) and anti-TH (a DA neuronal marker). To analyze dendrite length of neurons, cells were stained with anti-MAP2 (a dendritic marker) and anti-TH. The length of MAP2^+^ dendrite and Tuj1^+^ neurite in the neurons immuno-positive for TH was measured by NIH Image J with plugins simple neurite tracer.

### Isolation of subcellular fractions

Cells were washed with cold PBS and incubated on ice for 30 min in a lysis buffer (250 mM sucrose, 20 mM HEPES–NaOH, pH 7.5, 10 mM KCl, 1.5 mM MgCl2, 1 mM EDTA, protease inhibitor cocktail and phosphatase inhibitor cocktail). Mouse brains were minced and homogenized in the lysis buffer and then placed on ice for 30 min. Collected cells or tissue were disrupted 20 times by repeated aspiration through a 25-gauge needle, followed by a 30-gauge needle. The homogenates were spun at 800 g for 10 min at 4 °C, and the resulting supernatants were spun at 10,000 g for 20 min at 4 °C. The pellets were washed with lysis buffer and spun at 10,000 g again for 20 min at 4 °C. The final pellets were suspended in lysis buffer containing 1% Triton X-100 and were mitochondrial-rich lysate fractions. The supernatant was cytosolic fractions. The mitochondrial proteins VDAC, the ER protein WFS1, and the cytosolic protein Enolase were used as loading controls for mitochondrial, ER, and cytosolic fractions, respectively. The mitochondrial-rich lysate fractions were then spun at 10,000 g for 20 min and the supernatant was triton-soluble mitochondrial fraction. The pellets were washed twice using mitochondrial lysis buffer containing 1% Triton X-100 and suspended in the lysis buffer containing 1% Triton X-100 and 1% SDS, followed by incubation at 100 °C for 5 min. The final solution was triton-insoluble mitochondrial fraction.

### Preparation of triton-soluble and -insoluble fraction

Cells were washed with cold PBS and incubated on ice for 30 min in total cell lysate buffer (50 mM Tris–HCl, pH 7.5, 150 mM NaCl, 1% Triton X-100, and protease inhibitor). Collected cells were spun at 12,000 rpm for 10 min at 4 °C; the resulting supernatant was triton-soluble fraction. The pellet was further suspended in total lysis buffer with 1% SDS, and incubated at 100 °C for 3 min, and followed by 20-s vortex. After two more repeats of 100 °C incubation and vortex, the solution was spun at 12,000 rpm for 10 min at 4 °C; the resulting supernatant was triton-insoluble fraction.

### Preparation of digitonin-soluble and -insoluble fraction

Cells were washed with cold PBS and incubated on ice for 30 min in lysis buffer (50 mM Tris pH 7.4, 150 mM NaCl, protease inhibitor cocktail) containing 0.5% or 1% digitonin. Mouse brains were minced and homogenized in the lysis buffer and then placed on ice for 30 min. Collected cells or tissue were spun at 20,000 g for 20 min at 4 °C; the resulting supernatant was digitonin-soluble fraction. The pellet was further suspended in digitonin lysis buffer with 1% SDS, and incubated at 100 °C for 3 min, and followed by 20-s vortex. After two more repeats of 100 °C incubation and vortex, the solution was spun at 20,000 g for 20 min at 4 °C; the resulting supernatant was digitonin-insoluble fraction.

### In vitro fractionation of soluble and insoluble proteins

Recombinant ClpP protein is a gift from Dr. Aaron Schimmer (Princes Margartet Cancer Centre, Canada), and recombinant WT and A53T α-synuclein were purchased from rPeptide. Recombinant ClpP protein (0.5 mg/ml) was incubated with either WT or A53T α-synuclein or protein CHCHD3 (3 mg/ml) in PBS buffer containing 20 mM NaPO_4_ pH 7.4, 140 mM NaCl, at 37 °C for 8 h. Insoluble aggregates were separated by centrifugation at 10,000 g for 10 min. The supernatant containing soluble protein was transferred to a fresh Eppendorf tube. The pellet fraction was resuspended in PBS and centrifuged twice more. The pellet fraction was finally resuspended in PBS containing 1% SDS.

### Constructs and transfection

GFP-tagged αSyn-WT and -A53T plasmids (#40822, #40823), pBI-EGFP-MnSOD (#16612) were obtained from Addgene. To construct Myc-tagged αSyn-WT and –A53T, pCMV-Myc was digested with SalI and KpnI, and αSyn-WT or –A53T was PCR-amplified and inserted into the plasmid backbone. To construct Myc-tagged ClpP plasmid, pCMV-Myc was digested with EcoR1 and Xho1, and ClpP was PCR-amplified and inserted into the plasmid backbone. ClpP point mutant (S153A) was constructed using QuickChange II Site-Directed Mutagenesis Kit (200524, Agilent Technology). Cells were transfected with *Trans*IT-2020 (Mirus Bio, LLC) following the manufacturer’s protocol.

To construct AAV-ClpP, human ClpP cDNA was inserted into the plasmid backbone AAV5.hSyn.eGFP.WPRE.bGH (Cat# AV-5-PV1696) which was obtained from Penn Vector Core, University of Pennsylvania. AAV-GFP-ClpP and AAV-GFP control were then packed to obtain AAVs in the same core facility.

To construct Lenti-ClpP, human ClpP cDNA was inserted into the plasmid backbone pHR-IG (pHR’tripCMV-IRES-eGFP) (53597, Addgene). Lenti-ClpP and lenti-control were then packed to obtain lentivirus for the infection as previously described [35].

### Measurement of cell viability

SH-SY5Y cells were transfected with human ClpP siRNA or control siRNA for 3 days. After then, the cells were cultured in FBS-free DMEM/F12 (1:1) medium for 24 h. Neurons derived from iPS cells carrying αSyn A53T and its corrected isogenic control were cultured in neuronal differentiation medium (GDNF, ascorbic acid, TGFβ, cAMP) without BDNF for 24 h. Cell death was determined by measuring LDH release into the culture medium, using LDH-Cytotoxicity Assay Kit II (Roche, USA, 04744926001) by following the manufacturer’s instruction.

### RNA interference

For silencing ClpP in cells, control siRNA and ClpP siRNA were purchased from Thermo Fisher Scientific. HEK293, HeLa and SH-SY5Y cells were transfected either with control siRNA or Clpp siRNA using *Trans*IT-TKO Transfection Reagent (Mirus Bio, LLC, MIR 2154), according to the manufacturer’s instructions. The sequences for the siRNAs used in this study are as follows: ClpP siRNA, 5′-AAACAGAGCCUGCAGGUGA-3′; non-targeting control siRNA, 5′-TTCTCCGAACGTGTCACGT-3′.

### ClpP peptidase activity

Human recombinant ClpP (10 µM, obtained from Dr. Aaron Schimmer, Princes Margartet Cancer Centre, Canada) was incubated in the reaction buffer (50 mM Tris pH 8.0, 200 mM KCl, 1 mM DTT, 2 mM ATP) under 37 °C for 10 min. For co-incubation with α-synuclein, recombinant ClpP and αSyn-WT or -A53T were pre-incubated for 30 min under room temperature. Fluorescent substrate of ClpP, ac-WLA-AMC (50 µM), was then added in the reaction buffer. The fluorescence signal was read using TECAN infinite M1000 up for 30 min at excitation/emission wavelength 345/445. ClpP peptidase activity was determined as the slope of the regression line.

### Measurement of the amount of mitochondrial misfolded/unfolded proteins

Mitochondrial fractions were isolated as described above. Isolated mitochondria were suspended in PBS with 1% Triton X-100. A mixture of 40 µg mitochondrial protein and 50 µM TPE-MI dye (obtained from Dr. Yuning Hong, La Trobe University, Australia) in a final volume of 50 µl was added into a 96-well plate for fluorescence reading up to 2 h. The load of mitochondrial unfolded protein was represented as the highest relative fluorescence unit (RFU) in each group, as described in [15].

### Measurement of mitochondrial protein oxidation

Mitochondrial fractions were isolated and prepared as described above. 40 µg of mitochondrial protein in each group was subjected to protein oxidation determination using OxyBlot™ Protein Oxidation Detection Kit (Millipore, S7150) by following the manufacturer’s instruction.

### Measurement of mitochondrial respiratory activity

The SH-SY5Y cells, iPS cell colonies or neurons derived from patient iPS cells or isogenic control were seeded in XFp 8-well miniplates at 2000 cells per well in 50 µl of growth medium. Three days after transfection of ClpP siRNA or control siRNA, mitochondrial respiration activity in intact cells was analyzed using a Seahorse Bioscience XFp Extracellular Flux Analyzer. Briefly, 1 h prior to measuring oxygen consumption, the cell culture media were replaced with XF assay medium and maintained in a non-CO_2_ incubator for 1 h. The sensor cartridges were placed in the XFp Analyzer according to the manufacturer’s instructions for the Mito Stress Test kit. Mitochondrial function was determined by the sequential injection of oligomycin A (1 µM), FCCP (1 µM) and antimycin A (0.5 µM).

### Mitochondrial ROS measurement

Cells cultured on coverslips or 24-well plates were washed with PBS and then incubated with 5 µM MitoSOX™ Red (Invitrogen, M36008), a mitochondrial superoxide indicator, for 10 min at 37 °C. For cells cultured on coverslips, the images were visualized by microscope, and quantification of images was then carried out using NIH ImageJ software. At least 100 cells per group were counted in the analysis. For cells cultured on 24-well plates, MitoSOX intensity was measured (510 nm excitation/580 nm emission) by infinite M1000 multimode fluorescence plate reader (Tecan, Switzerland). The MitoSOX fluorescence density was normalized to the fluorescence density of DAPI (355 nm excitation/460 nm emission) which stained the nuclei.

### RT-PCR

Total RNA was isolated using RNeasy Mini Kit (QIAGEN), and 0.5–1 µg of total RNA was used to synthesize cDNA using QuantiTect Reverse Transcription Kit (QIAGEN). qRT-PCR was performed using QuantiTect SYBR Green (QIAGEN) and analyzed with the StepOnePlus Real-Time PCR System (Thermo Fisher Scientific). Three replicates were performed for each biological sample, and the expression values of each replicate were normalized against GAPDH cDNA using the 2^−ΔΔCT^method. The primers used were as follows: ClpP forward 5′-TTGCCAGCCTTGTTATCGCA-3′, ClpP Reverse 5′-GGTTGAGGATGTACTGCATCG-3′; GAPDH forward 5′-GGAGCGAGATCCCTCCAAAAT-3′, GAPDH reverse 5′-GGCTGTTGTCATACTTCTCATGG-3′.

### Co-immunoprecipitation

Cells were lysed in a total cell lysate buffer (50 mM Tris–HCl, pH 7.5, 150 mM NaCl, 1% Triton X-100, and protease inhibitor). Total lysates were incubated with the indicated antibodies overnight at 4 °C followed by the addition of protein A/G beads for 2 h at 4 °C.

Recombinant α-Synuclein and Clpp (500 ng) were incubated in in vitro interaction buffer (20 mM Tris–HCl pH 7.5, 100 mM KCl, 2 mM MgCl_2_ and 0.1% Triton-X100) for 30 min at room temperature, and then incubated with indicated antibodies overnight at 4 °C followed by the addition of protein A/G beads for 2 h. Immunoprecipitates were washed four times with cell lysate buffer/in vitro interaction buffer in the presence of 0.1% Triton X-100 and were analyzed by western blot analysis.

### Western blot analysis

Protein concentrations were determined by Bradford assay. Protein was resuspended in Laemmli buffer, loaded on SDS–PAGE, and transferred onto nitrocellulose membranes. Membranes were probed with the indicated antibodies, followed by visualization with ECL.

### Animal model of PD

All animal experiments were conducted in accordance with protocols approved by the Institutional Animal Care and Use Committee of Case Western Reserve University and were performed based on the National Institutes of Health Guide for the Care and Use of Laboratory Animals. Sufficient procedures were employed for reduction of pain or discomfort of subjects during the experiments.

αSyn A53T [B6.Cg-Tg(Prnp-SNCA*A53T)23Mkle/J, JAX Stock No: 006823] breeders (C57Bl/6J genetic background) were purchased from Jackson Laboratories. The mice were mated, bred, and genotyped in the animal facility of Case Western Reserve University. Male mice at the ages of 4, 6, 8, and 10 months were used in the study. All mice were maintained at a 12-h light/dark cycle (on 6 am, off 6 pm).

### Stereotaxic injection

Stereotaxic surgery was performed using a model 1900 stereotax (Kopf) under isoflurane anesthesia. Briefly, a small craniotomy was made using a 33-gauge drill bit above the desired coordinate. A small pulled glass pipette containing AAV was attached to a Nanoject II (Drummond) and was then inserted to the appropriate depth. Injections were performed at a rate of 90 nl/min. The coordinates used for substantia nigra injections were anteroposterior, − 3.0 mm from bregma; mediolateral, 1.2 mm; dorsoventral, 4.3 mm). The concentration (ddTiter) of the virus is 6.49e13 GC/ml. A volume of 200 nL of AAV5.hSyn.eGFP.ClpP.WPRE.bGH or AAV5.hSyn.eGFP.WPRE.bGH was injected into two hemispheres of mice. As a result, the number of viral particles of AAV is 1.298e7.

### Immunohistochemistry analysis

Mice were deeply anesthetized and transcardially perfused with 4% paraformaldehyde in PBS. Frozen brain sections (10 μm, coronal) were used for immunofluorescence staining of ClpP (1:200, Novus, NBP1-89557), TH (1:1000, Milliplore, MAB318), αSyn pS129 (1:10,000, BioLengend, 825701). For histochemical analysis of postmortem brains of normal subjects and PD patients, paraffin-embedded sections (5 µm) were stained with anti-ClpP and/or anti-TH antibodies. Quantitation of immunostaining was conducted using NIH image J software. The same image exposure times and threshold settings were used for sections from all groups. An experimenter blinded to the experimental groups conducted the quantitation.

### Behavioral analysis in PD mice

All behavioral analyses were conducted by an experimenter who was blinded to genotypes and treatment groups. Open-field locomotion movement activity was assessed in αSyn A53T mice and age-matched wild-type littermates at the age of 6 months prior to AAV injection, and the age of 7–10 months after AAV injection (once per month). The mice were placed in the center of an open-field chamber (Omnitech Electronics, Inc) and allowed to explore while being tracked by an automated beam system (Vertax, Omnitech Electronics Inc). A 24-h locomotor activity analysis was performed. The body weight and survival rate of αSyn A53T mice and wild-type littermates were recorded throughout the study period.

### Statistical analysis

Sample sizes were determined by a power analysis based on pilot data collected by our labs or published studies. In the cell culture studies, we performed each study with at least three independent replications. For all of the animal studies, we ensured randomization and blinded conduct in experiments. For all imaging analyses, an observer who was blind to the experimental groups conducted the quantitation.

Data were analyzed by Student’s *t* test or one-way ANOVA with post hoc Tukey’s test for comparison of multiple groups. Data are expressed as mean ± SEM. Statistical significance was considered achieved when the value of *p* was < 0.05.

## Results

### ClpP selectively decreases in cells expressing αSyn WT and A53T mutant

**Fig. 1:**
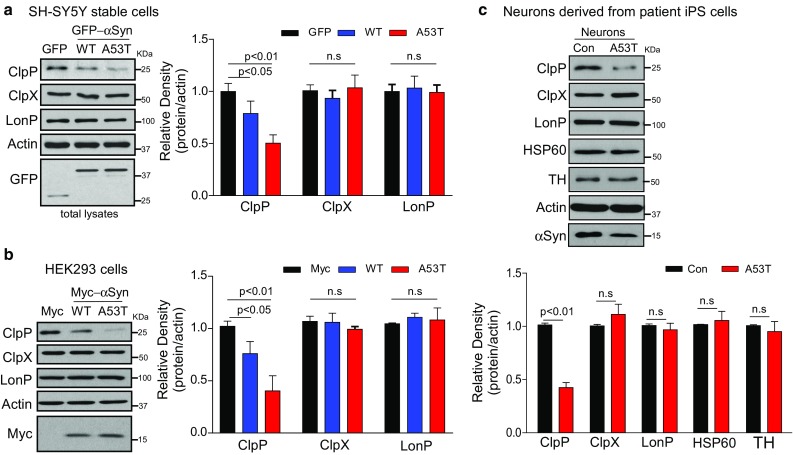
ClpP selectively decreases in cell cultures of αSyn-related PD. **a** Total lysates of SH-SY5Y cells stably expressing GFP control vector (GFP), GFP-αSyn WT (WT), or GFP-αSyn A53T (A53T) were subjected to western blot analysis with the indicated antibodies. Quantitative analysis of protein expression levels was performed by intensity measurement of ClpP, ClpX and LonP in contrast to β-actin. One-way ANOVA with Tukey’s post hoc test. **b** Total lysates of HEK293 cells overexpressing Myc control vector (Myc), Myc-αSyn WT (WT) or Myc-αSyn A53T (A53T) for 2 days were subjected to western blot analysis with the indicated antibodies. Intensity quantitation of ClpP, ClpX or LonP relative to β-actin is shown in the histogram. One-way ANOVA with Tukey’s post hoc test. **c** Neuronal cells were differentiated from iPS cells of PD patient carrying αSyn A53T mutant and isogenic corrected control for 40 days. Total cell lysates from the mixture of neuronal cells were harvested and subjected to western blot analysis with the indicated antibodies. Histogram: quantitative data (protein density versus β-actin) were averaged from three independent differentiation batches. The quantitative data from each of independent differentiation batch are shown in Suppl Fig. 1e. The Student’s *t* test. All data are expressed as mean ± SEM of at least three independent experiments

Consistent with previous studies [23, 51], we found that αSyn was present in the mitochondria of dopaminergic SH-SY5Y cells stably expressing GFP-αSyn Wildtype (WT) or A53T mutant (A53T) (Suppl Fig. 1a). While αSyn WT and A53T mutant appeared in the detergent-insoluble mitochondrial fractions, suggestive of aggregation, the A53T mutant exhibited greater mitochondrial aggregation in the detergent-insoluble fraction in SH-SY5Y cells (Suppl Fig. 1a). The purity of mitochondrial fractions was validated by western blot analysis (Suppl Fig. 1b). After removing mitochondrial outer membrane by incubation of the mitochondrial fractions with proteinase K, we further demonstrated that Myc-αSyn WT and Myc-αSyn A53T mutant were accumulated inside the mitochondria of cells (Suppl Fig. 1c). Note that the total level of αSyn WT and αSyn A53T was comparable (Fig. 1a, b).

To examine whether ClpP is affected by αSyn WT or its mutant A53T, we first determined the protein level of ClpP in dopaminergic SH-SY5Y cells stably expressing GFP-αSyn WT or A53T. Western blot analysis revealed that stable expression of αSyn WT or A53T mutant in SH-SY5Y cells decreased the protein level of ClpP, but led to an even greater reduction in GFP-αSyn A53T-expressing cells (Fig. 1a). Similarly, the protein level of ClpP was significantly lower in HEK293 cells transiently overexpressing Myc-αSyn WT or Myc-αSyn A53T, relative to cells with Myc control vector (Fig. 1b). In contrast, expression of either αSyn WT or A53T had no effect on the mitochondrial matrix protease LonP or the ClpP ATP-binding subunit ClpX-like (ClpX, an essential component of ClpXP protease complex [44]) in the above cell cultures (Fig. 1a, b). The level of ClpP mRNA was not altered in αSyn WT and A53T stable SH-SY5Y cells (Suppl Fig. 1d), suggesting a transcriptional-independent effect.

We next differentiated induced pluripotent stem (iPS) cells from PD patient carrying αSyn A53T and its isogenic corrected control into neuronal cells. The neuronal cells derived from αSyn A53T PD patient iPS cells exhibited an accumulation of αSyn serine 129 phosphorylation (αSyn-pS129), a specific pathological αSyn form [2], and shorter neurites of dopaminergic neurons immunopositive for anti-tyrosine hydroxylase (TH), relative to neurons derived from isogenic corrected control iPS cells (Suppl Fig. 2). We found that not only in iPS cells of αSyn A53T PD patient (Suppl Fig. 2d) but also in neurons derived from iPS cells of PD patient carrying αSyn A53T mutant (Fig. 1c), the protein level of ClpP was decreased when compared to that in isogenic corrected iPS cells and their derived neuronal culture, respectively (Suppl Figs. 2d, 1c). The protein levels of LonP, ClpX and HSP60 were not changed (Suppl Figs. 2d, 1c). The mRNA level of ClpP in neurons derived from αSyn A53T patient iPS cells and isogenic control was comparable (Suppl Fig. 2e). Note that we tested the change of ClpP in one line of αSyn A53T PD patient iPS cell and its isogenic control, which are only available to us. Future study using multiple lines of PD patient iPS cells may warrant a better understanding of the change of ClpP in PD patient neurons.

All together, these findings demonstrate a selective decrease in ClpP protein level in cell culture by αSyn WT and A53T mutant both exogenously and endogenously.

### ClpP selectively decreases in DA neurons of αSyn A53T mice and PD patients

**Fig. 2:**
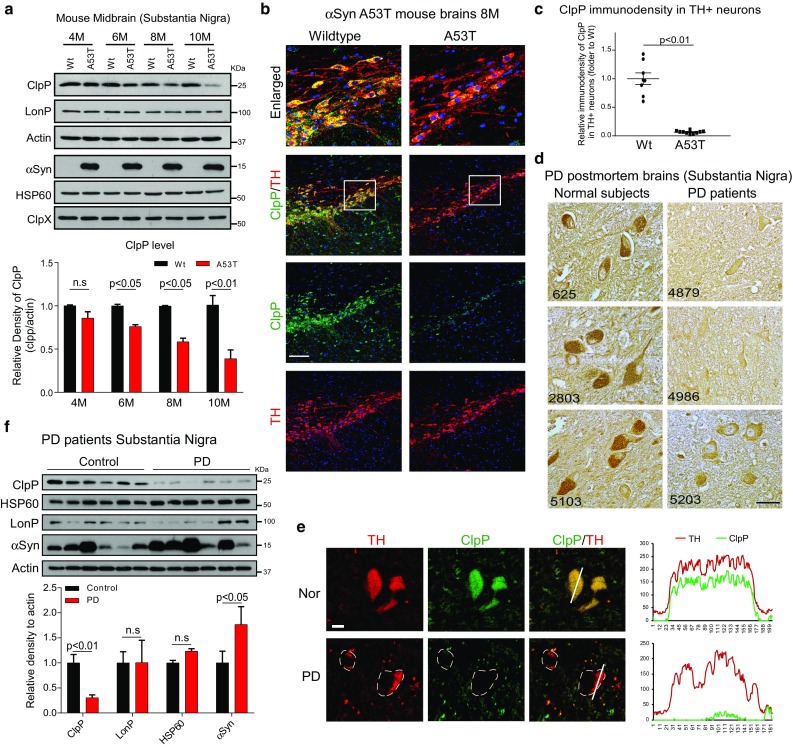
ClpP selectively decreases in DA neurons of αSyn A53T mice and PD patient postmortem brains. **a** The midbrains containing the Substantia nigra (SN) were harvested at the ages of 4, 6, 8, and 10 months of wildtype littermates (Wt) and αSyn A53T (A53T) mice. Total protein levels of ClpP, LonP, HSP60, ClpX and αSyn were examined by western blot analysis. *n* = 4 mice/group. Histogram: quantitation of ClpP protein intensity in contrast to actin. Data are expressed as mean ± SEM. ANOVA with Tukey’s post hoc test. **b** Brain sections of Wt and αSyn A53T mice at the age of 8 months were stained with anti-ClpP (green) and anti-TH (red) antibodies. *n* = 3 mice/group. Scale bar: 10 µm. The top panel shows enlarged images of boxed area. **c** Quantification of the localization of ClpP in TH^+^ neurons in (B) was shown. Data are expressed as mean ± SEM. Student’s *t* test. **d** Postmortem SN brain sections of normal subject (ID: 625, 2803, 5103) and PD patients (ID: 4879, 4986, 5203) were stained with anti-ClpP antibodies. The information of human samples was listed in Suppl Fig. 3f. Scale bar: 10 µm. **e** The SN sections of postmortem brains of normal subjects and PD patients were stained with anti-ClpP (green) and anti-TH (red) antibodies. Line profile was used to illustrate co-localization between ClpP and TH. Green and red lines indicate ClpP and TH staining profiles, respectively. Scale bar: 10 µm. **f** Total protein lysates were obtained from the frozen SN of six PD patients and six control subjects. Western blot analysis was carried out with the indicated antibodies. Histogram: quantitation of protein intensity in contrast to actin. Data are expressed as mean ± SEM. Student’s *t* test. The information on PD patients and control subjects is presented in Suppl Fig. 3i

Next, we assessed the change of ClpP in αSyn PD mice in vivo. We used Hu-αSyn (A53T) transgenic mice [also called G2-3(A53T)] [16], in which human αSyn A53T transgene is driven by the mouse prion promoter [47]. The mice spontaneously develop adult-onset progressive motor deficits, exhibit typical αSyn pathology including αSyn-pS129 and the formation of αSyn fibrils, and present markers of mitochondrial oxidative damage [10, 47, 53]. We harvested total protein lysates from midbrain containing substantia nigra (SN), cortex, striatum and brainstem of αSyn A53T mice or wild-type (Wt) mice at the ages of 4, 6, 8 and 10 months. Western blot analysis showed that the protein level of ClpP significantly decreased in the midbrain of 6-month-old αSyn A53T mice. The nigral expression of ClpP which continued to decrease with age was found to be substantially reduced in 10-month-old αSyn A53T mice compared to the age-matched Wt mice (Fig. 2a). The protein levels of mitochondrial matrix protease LonP, ClpX and HSP60 were not altered between αSyn A53T and Wt mice at any of the ages examined (Fig. 2a), excluding the possibility that the reduction of ClpP results from the loss of mitochondrial mass in vivo. In contrast, levels of ClpP between αSyn A53T and Wt mice were comparable in striatum, cortex and brainstem (Suppl Fig. 3a). Note that αSyn A53T was accumulated in the mitochondria of mouse SN (Suppl Fig. 3b). The mRNA level of ClpP was comparable between Wt and A53T mice at the age of 10 months (Suppl Fig. 3c).

Immunohistochemical analysis consistently found that expression of ClpP was substantially reduced in the neurons immunopositive for anti-Tyrosine Hydroxylase (TH, a DA neuronal marker) in the SN of αSyn A53T mice relative to that in age-matched Wt mice (8 months old), whereas the levels of TH in the SN were comparable between Wt and αSyn A53T mice (Fig. 2b, c, Suppl Fig. 3d). No change in ClpP immunodensity was observed in the cortex of αSyn A53T mice at the same age (Suppl Fig. 3e). Further, a clear decrease in ClpP immunodensity was observed in the SN of three PD patients when compared to those of age-matched normal subjects (Fig. 2d, see Suppl Fig. 3f on the information of human samples). Double immunofluorescence staining confirmed a reduction of ClpP expression in the remaining DA neurons immunopositive for anti-TH antibodies in the SN of PD patient postmortem brain (Fig. 2e). Note that all three PD patients exhibited αSyn-enriched aggregates (Suppl Fig. 3g). Western blot analysis consistently showed that the protein level of ClpP was lower in the postmortem SN of 6 PD patients than that in control subjects (Fig. 2f, see Suppl Fig. 3i on the information of human samples). In contrast, the level of ClpP in the cortex of both control subjects and PD patients was comparable (Suppl Fig. 3h). Consistent with previous studies [16, 18, 27, 43], the protein levels of αSyn in both the SN and cortex of PD patients were higher than that in control subjects (Fig. 2f, Suppl Fig. 3h). These results support our observations in cell culture and demonstrate a decrease of ClpP in DA neurons of SN in both PD mice and PD patient brains.

### A deficiency in ClpP induces mitochondrial bioenergetic defects and oxidative damage

**Fig. 3:**
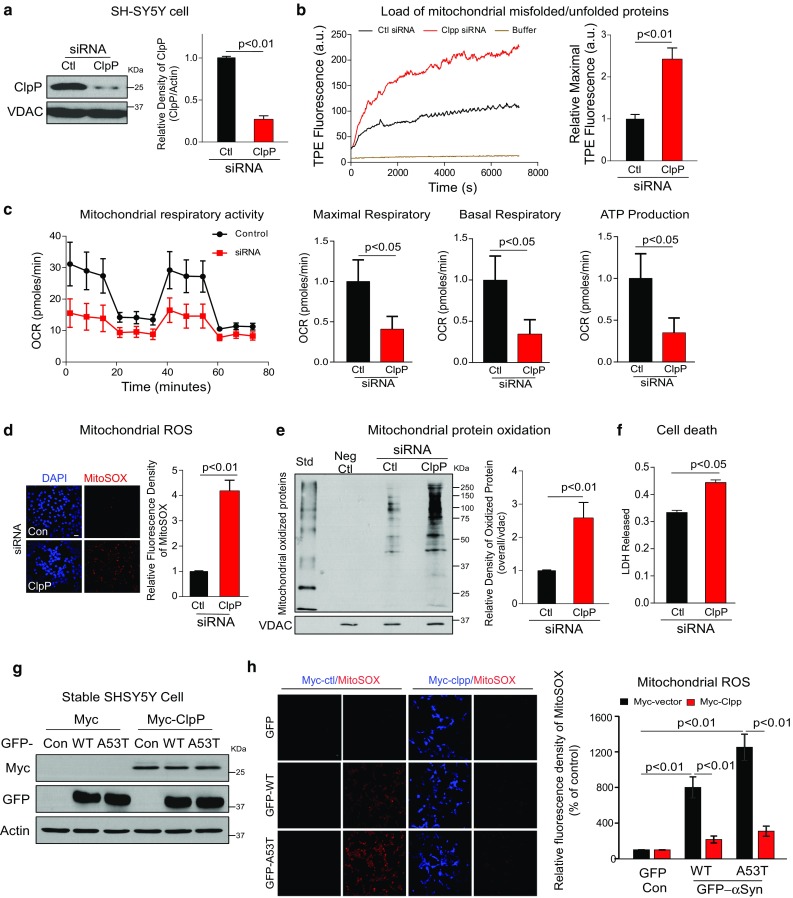
ClpP deficiency induces mitochondrial bioenergetic defect and oxidative damage. ClpP was knocked down by ClpP siRNA for 3 days in SH-SY5Y cells. **a** ClpP knockdown efficiency was examined by western blot analysis. Student’s *t* test. **b** Mitochondria of SH-SY5Y cells were isolated after ClpP silencing and incubated with TPE-MI dye, followed by measurement of fluorescence intensity for 2 h. Histogram: Maximal fluorescence was used to reflect the loading of mitochondrial unfolded proteins. Student’s *t* test. **c** Mitochondrial respiratory activity was measured using seahorse analyzer. Maximal OCR, basal OCR and ATP content were calculated and shown in histograms. Student’s *t* test. **d** Mitochondrial superoxide production was determined using mitochondrial superoxide indicator, MitoSOX™ red, in the indicated groups. Left: representative images. Scale bar: 10 µm. Right: quantitation of fluorescence density of MitoSOX red/cell. At least 100 cells/per group were counted. Student’s *t* test. **e** Mitochondrial protein oxidation was determined. *Std* standard marker, *Neg Ctl* negative control. Shown blots are representative of three independent experiments. Histogram shows the intensity measurement of oxidized proteins in contrast to mitochondrial loading control VDAC. Student’s *t* test. **f** Cell death was determined by measuring the LDH release into medium. Student’s *t* test. **g** Stable SH-SY5Y cells were transfected with Myc control vector (Myc) or Myc-ClpP, and **h** mitochondrial superoxide production was determined using mitochondrial superoxide indicator, MitoSOX™ red, in the indicated groups. Left: representative images. Right: quantitation of fluorescence density of MitoSOX red/cell. Scale bar: 10 µm. At least 100 cells/per group were counted. Two-way ANOVA with Tukey’s posthoc test. All data are expressed as mean ± SEM of at least three independent experiments

To determine if loss of ClpP affects mitochondrial function and integrity, we down-regulated ClpP in dopaminergic SH-SY5Y neuronal cells by RNA interference (siRNA) (Fig. 3a). Given that ClpP is required for protein turnover in the mitochondrial matrix and is upregulated during UPR^mt^ in response to stress [38], we first assessed whether the loss of ClpP leads to protein misfolding in the mitochondria. Accumulated unfolded/misfolded proteins in subcellular organelles can be detected and quantitated by tetraphenylethene maleimide (TPE-MI) fluorescence dye [15]. TPE-MI fluorescence is activated only upon labeling free cysteine thiols, normally buried in the core of globular proteins that are exposed upon unfolding [15]. We found an increase in the amount of TPE-MI fluorescence-labeled unfolded proteins in the mitochondrial fractions isolated from SH-SY5Y cells in the presence of ClpP siRNA compared to that in cells with control siRNA (Fig. 3b), suggesting that the function of ClpP in proteostasis maintenance is conserved across mammals, *C*-*elegans* and bacteria [38].

Notably, down-regulation of ClpP by siRNA strongly suppressed mitochondrial respiratory activity in dopaminergic SH-SY5Y cells, as demonstrated by the findings that ClpP silencing reduced mitochondrial maximal oxygen consumption rate, basal oxygen consumption rate and ATP content in the intact cells (Fig. 3c). In parallel, knockdown of ClpP increased mitochondrial superoxide production (mitoROS) (Fig. 3d), coincident with an increase in the amount of oxidized mitochondrial proteins (Fig. 3e). SH-SY5Y cells stably expressing αSyn, especially A53T mutant, exhibited decreased mitochondrial respiratory activity and increased load of oxidized proteins (Suppl Fig. 4a, b). Similarly, in both αSyn A53T PD patient iPS cells and neurons derived from αSyn A53T PD patient iPS cells, we observed impaired mitochondrial respiratory activity and increased mitochondrial oxidative stress (Suppl Fig. 4e, f). These mitochondrial malfunctions might mirror the decrease in ClpP protein level in these cultured cells (Fig. 1a, c, Suppl Fig. 2d). In addition, the reduction of ClpP by siRNA induced cell death which was measured by LDH release (Fig. 3f). Again, an increase in cell death rate was observed in neurons derived from αSyn A53T patient iPS cells (Suppl Fig. 4g), in which the protein level of ClpP was low (Fig. 1c). To test if gain-of-function of ClpP would rescue mitochondrial oxidative damage caused by αSyn, we overexpressed Myc-tagged ClpP (Myc-ClpP) or control vector in SH-SY5Y cells stably expressing αSyn WT or A53T mutant (Fig. 3g). Expression of either αSyn WT or A53T mutant elicited a strong increase in mitoROS, whereas overexpression of Myc-ClpP abolished this elevation (Fig. 3h). Our data, therefore, suggest that ClpP is required for the maintenance of mitochondrial protein homeostasis and bioenergetic activity in dopaminergic SH-SY5Y cells, and that a decrease in ClpP due to the presence of pathological αSyn causes mitochondrial oxidative damage.

### SOD2 is a downstream of ClpP in αSyn-expressing cells and αSyn PD mouse brains

Manganese Superoxide Dismutase (MnSOD/SOD2) resides predominantly in the mitochondrial matrix and functions primarily as a catalyst of superoxide radical dismutation to H_2_O_2_ [55, 77]. Recent studies report that SOD2 expression is elevated by the UPR^mt^ via SIRT3/FOXO3a-dependent transcription, by which it increases mitochondrial fitness and buffers ROS in the cells [46, 56, 64]. Moreover, SOD2 expression has been shown to correlate with the change of ClpP in mouse hepatocyte cell line exposed to stressors [56]. Given that genetic manipulation of ClpP alters mitochondrial superoxide production (Fig. 3), we hypothesize that SOD2 mediates ClpP-dependent mitochondrial oxidative damage in αSyn-associated PD models.

**Fig. 4:**
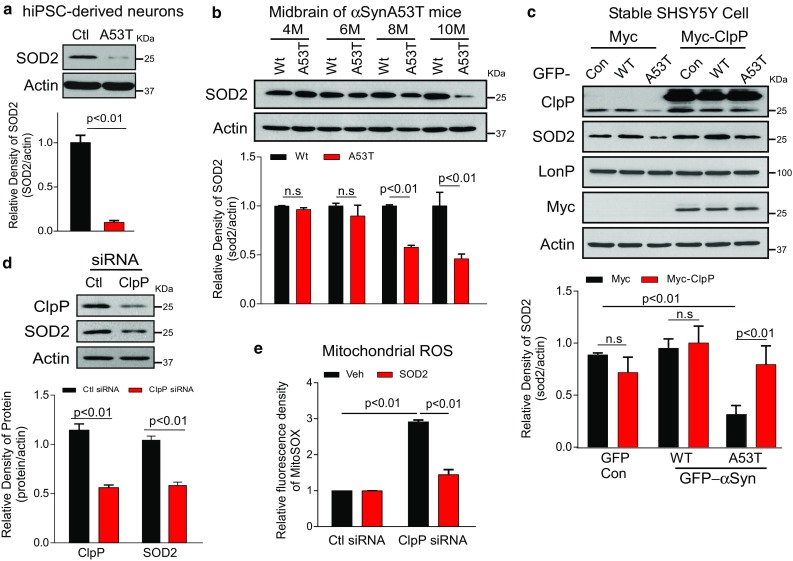
SOD2 is a downstream of ClpP. **a** Neuronal cells were differentiated from iPS cells of PD patient carrying αSyn A53T mutant or isogenic corrected control. Total lysates of cells were subjected to western blot analysis with the indicated antibodies. Histogram: the intensity measurement of SOD2 in contrast to actin. Student’s *t* test. **b** The midbrains containing the Substantia nigra (SN) of wildtype (Wt) and αSyn A53T (A53T) mice were harvested at the ages of 4, 6, 8, 10 months, respectively. The protein level of SOD2 was examined by western blot analysis. Histogram: the intensity measurement of SOD2 in contrast to actin. *n* = 4 mice/group. ANOVA with Tukey’s post hoc test. **c** Stable SH-SY5Y cells were transfected with Myc control vector (Myc) or Myc-tagged ClpP for 2 days. Total lysates of cells were subjected to western blot analysis with the indicated antibodies. Histogram: the intensity measurement of SOD2 in contrast to actin. Two-way ANOVA with Tukey’s post hoc test. **d** HEK293 cells were transfected with control (*Ctl*) or ClpP siRNA for 3 days. The protein levels of SOD2 and ClpP were examined by western blot analysis. Histogram: the intensity measurement of SOD2 and ClpP in contrast to actin. Student’s *t* test. **e** In the presence or absence of ClpP siRNA, HeLa cells were overexpressed with control vector or SOD2 plasmid for 48 h. Mitochondrial superoxide production was determined using mitochondrial superoxide indicator, MitoSOX™ red, by a fluorescence reader in the indicated groups. One-way ANOVA with Tukey’s post hoc test. All data are mean ± SEM of three independent experiments

Similar to ClpP, we observed a significant decrease in the protein levels of SOD2 in both αSyn A53T PD patient iPS cells (Suppl Fig. 5a) and neuronal cells derived from PD patient iPS cells carry αSyn A53T (Fig. 4a), and in the midbrain containing the SN of αSyn A53T mice starting from the age of 8 months (Fig. 4b). While αSyn A53T expression in SH-SY5Y cells reduced the level of SOD2, overexpression of Myc-ClpP corrected this decrease (Fig. 4c). Upon silencing ClpP in HEK293 cells, the protein level of SOD2 was correspondingly decreased (Fig. 4d). By knocking down ClpP in HEK293 cells using random doses of ClpP siRNA, we further observed a concomitant decrease of SOD2 level, exhibiting a positive correlation with ClpP (Suppl Fig. 5b, c). To further determine if ClpP deficiency-induced mitoROS is due to the decrease in SOD2 level, we knocked down ClpP in SH-SY5Y cells followed by overexpression of SOD2 construct. We found that increasing the level of SOD2 greatly attenuated mitoROS induced by silencing ClpP (Fig. 4e). These data collectively suggest that SOD2 and ClpP belong to the same signaling pathway, and SOD2 acts downstream of ClpP to elicit mitoROS in αSyn-associated PD models.

### αSyn promotes an aberrant distribution of ClpP from soluble to insoluble fractions in vitro and in vivo

**Fig. 5:**
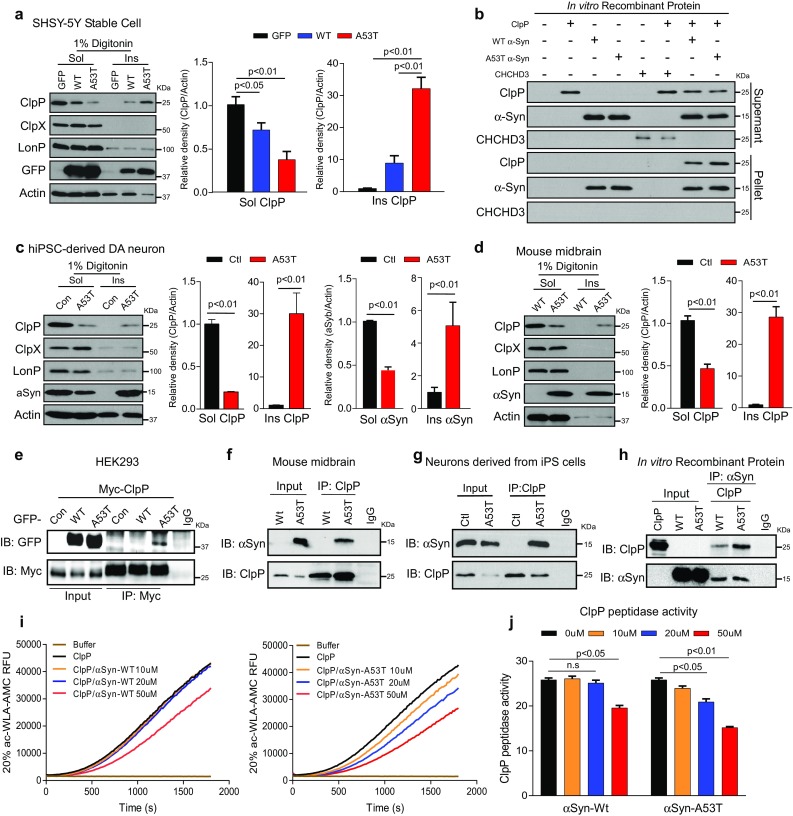
αSyn impairs ClpP proteolytic activity and promote its insolubility by interacting with ClpP. **a** Digitonin-soluble and -insoluble fractions were isolated from SH-SY5Y cells stably expressing GFP control vector (GFP), GFP-αSyn WT (WT), or GFP-αSyn A53T (A53T), and were then subjected to western blot analysis with the indicated antibodies. Quantitative analysis of protein expression levels was performed by intensity measurement of ClpP in contrast to β-actin. One-way ANOVA with Tukey’s post hoc test. **b** ClpP (10 µg) recombinant protein was incubated with αSyn WT or A53T mutant or CHCHD3 (50 µg, each) recombinant protein. Soluble supernatant and insoluble pellet were separated and subject to western blot analysis with the indicated antibodies. Shown blots are representative of three independent experiments. Digitonin-soluble and -insoluble fractions were isolated from **c** mixed neuronal culture differentiated from iPS cells of PD patient carrying αSyn A53T mutant or isogenic corrected control, and **d** the midbrain containing the Substantia nigra (SN) of αSyn A53T or wildtype mice. Western blot analysis was carried out with the indicated antibodies. Quantitative analysis of protein expression levels was performed by intensity measurement of ClpP in contrast to β-actin. Student’s *t* test. **e** HEK293 cells were transfected with GFP control (GFP), GFP-αSyn WT, or GFP-αSyn A53T for 2 days in the presence of Myc-ClpP. The total protein lysates in the presence of 1% Triton-X100 were subjected to immunoprecipitation (IP) with anti-Myc antibody, followed by immunoblotting (IB) with anti-GFP and anti-Myc antibodies. Shown blots are representative of three independent experiments. **f** The midbrains containing the Substantia nigra (SN) of wildtype (Wt) and αSyn A53T mice were harvested at the age of 10 months. Total protein lysates of mouse midbrains in the presence of 1% Triton-X100 were subjected to IP followed by IB with the indicated antibodies. *n* = 4 mice/group. Shown blots are representative. **g** Total lysates were harvested from mixed neuronal culture derived from iPS cells of αSyn A53T patient and isogenic corrected control, and subjected to IP followed by IB with the indicated antibodies. Shown blots are representative of three independent experiments. **h** ClpP recombinant protein (500 ng) was incubated with αSyn WT or αSyn A53T recombinant protein (500 ng, each) in the presence of 0.1% Triton-X100. Immunoprecipitates with anti-αSyn antibodies were analyzed by IB with anti-ClpP and anti-αSyn antibodies. Shown blots are representative of 3 independent experiments. **i** Left: ClpP recombinant protein (10 µM) was incubated with recombinant αSyn WT protein (10, 20 or 50 µM). The fluorescence intensity of ac-WLA-AMC (50 µM) was measured up to 30 min immediately after the addition. Right: ClpP recombinant protein (10 µM) was incubated with recombinant αSyn A53T (10, 20 or 50 µM). The fluorescence intensity of ac-WLA-AMC (50 µM) was measured up to 30 min immediately after the addition. **j** Quantification of ClpP peptidase activity after incubation with αSyn WT or A53T. One-way ANOVA with Tukey’s post hoc test. All data are mean ± SEM of three independent experiments

To investigate the effect of αSyn on ClpP cellular distribution and solubility, we lysated SH-SY5Y cells stably expressing αSyn WT or A53T mutant with different concentrations of digitonin, and separated into detergent-soluble and -insoluble fractions. Immunoblotting analysis showed that the protein level of ClpP in the detergent-insoluble fraction was increased in αSyn-expressing SH-SY5Y cells, with a higher extent in αSyn A53T mutant-expressing cells, whereas the level of ClpP in detergent-soluble fraction was correspondingly decreased (Fig. 5a, Suppl Fig. 6a). In contrast, the presence of αSyn WT or A53T mutant did not influence the distribution of both ClpX and LonP (Fig. 5a, Suppl Fig. 6a), suggesting a selectivity. To confirm a direct effect of αSyn on ClpP solubility, we incubated αSyn WT or A53T recombinant protein with ClpP recombinant protein in vitro, followed by a separation of soluble supernatant and insoluble pellet. The addition of αSyn WT or A53T mutant recombinant protein promoted an accumulation of ClpP in the insoluble pellet (Fig. 5b). Neither αSyn WT nor A53T mutant protein had effects on the distribution of CHCHD3, a mitochondrial inner-membrane protein with similar molecular weight as ClpP (25 kDa) (Fig. 5b).

We next examined whether αSyn accumulation in neurons of PD patients and mouse brains results in a similar alteration in ClpP distribution. Western blot analysis with detergent-insoluble and -soluble fractions showed that insoluble ClpP and αSyn accumulated, whereas soluble ClpP and αSyn decreased in neurons differentiated from αSyn A53T PD patient iPS cells when compared to that in neurons derived from isogenic corrected control (Fig. 5c). Similarly, we observed the accumulation of αSyn and ClpP in the insoluble fraction of the midbrain containing the SN of αSyn A53T mice (Fig. 5d, Suppl Fig. 6b). Collectively, our data in vitro and in vivo demonstrate that αSyn, especially, A53T mutant, promotes a translocation of ClpP from detergent-soluble to -insoluble fraction, which results in the reduced solubility of ClpP, leading to protein aggregation.

### αSyn interacts with ClpP and suppresses ClpP peptidase activity

αSyn can interact with a number of proteins to trigger neurotoxicity via initiation of downstream signals [41]. Given that the downregulation of ClpP by αSyn is not due to the transcriptional suppression and the solubility of ClpP alters in the presence of αSyn, we next asked if αSyn interacts with ClpP, by which to influence ClpP protein distribution and activity. After co-overexpressing Myc-ClpP and GFP-αSyn WT or A53T in HEK293 cells followed by co-immunoprecipitation (Co-IP), we found that Myc-ClpP strongly bound to GFP-αSyn A53T but not to GFP-αSyn WT (Fig. 5e). No bindings between ClpX and αSyn nor between LonP and αSyn were observed (Suppl Fig. 6c, d), suggesting a selective interaction. Co-IP analysis with the total protein lysates obtained from the midbrain of αSyn A53T mice also showed a strong interaction between αSyn and ClpP (Fig. 5f). Further, an endogenous binding between ClpP and αSyn was observed in the total lysates of neurons derived from αSyn A53T patient iPS cells but not in neurons derived from isogenic control iPS cells (Fig. 5g). These data exclude the possibility that elevated αSyn/ClpP binding is a result of αSyn overexpression in vitro and in vivo. To examine if ClpP and αSyn directly interact, we performed in vitro protein binding assay using recombinant human ClpP and αSyn WT or A53T proteins in the presence of 0.1% Triton to avoid non-specific binding. Co-IP analysis demonstrated that ClpP strongly interacted with αSyn A53T protein, while it weakly bound to αSyn WT protein (Fig. 5h). A future study on which state of αSyn, either monomer or oligomers, interacts with ClpP to lower ClpP solubility, is worthy.

ClpP is a mitochondrial serine protease, and its peptidase activity is required for degradation of mitochondrial misfolded/unfolded proteins [3, 39, 85]. We next incubated ClpP recombinant protein with αSyn WT or A53T mutant recombinant proteins followed by the addition of the ClpP fluorogenic substrate ac-WLA-AMC in ClpP peptidase assay buffer. The cleavage rate of ClpP fluorogenic substrate was determined to reflect ClpP peptidase activity with the method described [19] (Suppl Fig. 6e). We found that αSyn A53T significantly suppressed the peptidase activity of ClpP in a dose-dependent manner (Fig. 5i, j), whereas αSyn WT interfered with the peptidase activity of ClpP only at the highest dose (50 µM) used (Fig. 5i, j). Thus, αSyn A53T is more toxic than αSyn WT to the peptidase activity of ClpP, which is likely explained by the higher affinity of ClpP to αSyn A53T than αSyn WT. Taken together, these results support the possibility that interaction between αSyn and ClpP leads to the formation of insoluble aggregates that might result into reduced protein level of soluble ClpP in αSyn models in vitro and in vivo (Figs. 1, 2). This interaction also impairs the proteolytic activity of ClpP, which causes the overload of misfolded proteins in the mitochondria and subsequent mitochondrial damage. We found that neither downregulation of ClpP by siRNA nor overexpression of ClpP affected the endogenous protein level of αSyn in dopaminergic SH-SY5Y cells, though ERAL1, a known substrate of ClpP [83], changed correspondingly (Suppl Fig. 6f). The data exclude the possibility that αSyn is a substrate of ClpP.

### Overexpression of ClpP attenuates mitochondrial oxidative damage and pathological αSyn accumulation, and promotes neuronal morphology in neurons derived from αSyn A53T patient iPS cells

**Fig. 6:**
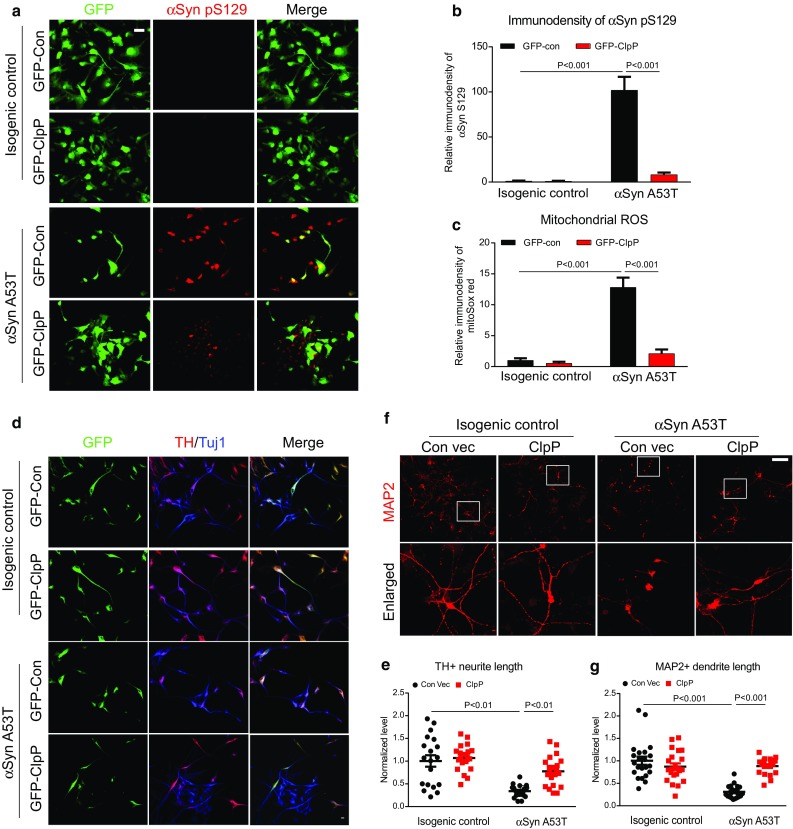
Overexpression of ClpP reduces αSyn phosphorylation and mitochondrial oxidative damage, and promotes morphology of neurons derived from iPS cells of αSyn A53T PD patient. Neuronal cells were differentiated from iPS cells of PD patient carrying αSyn A53T mutant or isogenic corrected control. 40 days after neuronal differentiation, cells were infected with lentivirus containing GFP control or GFP-labeled lentiviral ClpP. **a** Cells were stained with anti-αSyn pS129 (red). Scale bar: 10 µm. **b** The immunodensity of αSyn-pS129 in GFP-positive cells was quantitated. **c** Cells were stained with mitoSOX red and fluorescence density was quantitated to indicate mitochondrial superoxide production. At least 50 neurons/group were counted. **d** Cells were stained with anti-TH (red) and anti-beta-Tubulin III (Tuj1, blue). Scale bar: 10 µm. **e** The length of neurites in TH^+^/Tuj^1+^/GFP^+^ cells was quantitated. At least 20 neurons/group were analyzed. **f** Cells were stained with anti-MAP2. Scale bar: 10 µm. **g** The length of neurites in MAP^2+^ cells was quantitated. At least 20 neurons/group were analyzed. All data are mean ± SEM from three independent experiments. One-way ANOVA with Tukey’s post hoc test

Phosphorylation of αSyn at Ser129 promotes the accumulation of oligomeric αSyn [2], accelerates the formation of αSyn aggregations in neurons [79], and triggers neuronal loss in mice [13, 87]. A high level of αSyn-pS129 was recently found to be in close proximity to fragmented mitochondrial membranes in DA neurons derived from iPS cells of PD patients carrying αSyn A53T, whereas no enrichment of pS129-modified αSyn was identified on ER or Golgi membranes [73]. Thus, αSyn-pS129 can be used as a marker to assess the pathological form of αSyn preferentially associated with dysfunctional mitochondria. In neurons derived from αSyn A53T PD patient iPS cells, consistent with the previous study [73], we observed an accumulation of αSyn-pS129, which was not detected in neurons differentiated from isogenic corrected control iPS cells (Fig. 6a, b, Suppl Fig. 2). In contrast, expression of GFP-tagged lentiviral ClpP greatly reduced the amount of αSyn-pS129 in neurons derived from PD patients (Fig. 6a, b). Moreover, the expression of ClpP attenuated mitochondrial oxidative damage (Fig. 6c), and promoted the neurite length of TH^+^/Tuj1+ neurons when compared to those in neurons infected with control vector (Fig. 6d, e). In addition, overexpression of ClpP improved MAP2^+^ dendrite length of neurons derived from αSyn-A53T patient iPS cells (Fig. 6f, g). No effect of ClpP expression on morphology of neurons derived from isogenic corrected control iPS cells was observed (Fig. 6f, g).

### Viral expression of ClpP reduces αSyn-associated neuropathology in αSyn A53T mice

**Fig. 7:**
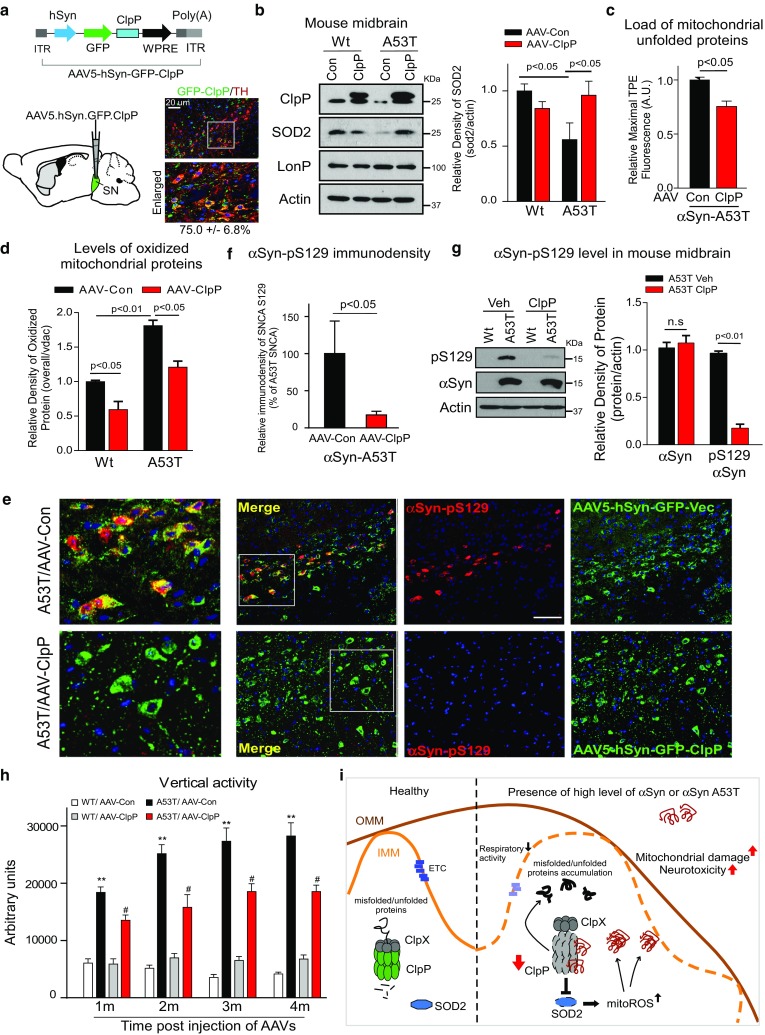
Viral expression of ClpP reduces mitochondrial damage and pathology in αSyn A53T mice. **a** Stereotaxical injection of AAV5-hSyn-eGFP-ClpP into the substantia nigra (SN) of mice. AAV induced expression of GFP in the SN 2 months after injection. Image: DA neurons in the SN were marked by anti-TH antibodies (red). The transduction efficiency of AAV in TH^+^ DA1 neurons is 75.0 ± 6.8%, shown at the bottom of the image. Scale bar is 20 µm. The midbrains containing the Substantia nigra (SN) of wildtype (Wt) and αSyn A53T (A53T) mice were harvested 4 months after injection of AAV-control vector or AAV-GFP-ClpP. **b** Total lysates of mouse midbrains were subjected to western blot analysis with the indicated antibodies. *n* = 4 mice/group. Histogram: the intensity measurement of SOD2 in contrast to actin. One-way ANOVA with Tukey’s post hoc test. **c** Mitochondria were isolated from the midbrains of mice. Load of mitochondrial misfolded/unfolded proteins was examined using TPE-MI dye. *n* = 4 mice/group. Student’s *t* test. **d** Mitochondrial protein oxidation was analyzed using protein oxidation kit. *n* = 4 mice/group. One-way ANOVA with Tukey’s post hoc test. **e** Brain sections of αSyn A53T mice were stained with anti-αSyn pS129 (red) and Hoescht (blue). *n* = 3 mice/group The left panel shows enlarged image of boxed area. Scale bar: 10 µm. **f** Histogram shows the quantitation of αSyn-pS129 immunodensity. Student’s *t* test. **g** Total protein lysates of mouse midbrains were subjected to Western blot analysis with the indicated antibodies. *n* = 4 mice/group. Histogram: the intensity measurement of αSyn-pS129 in contrast to actin. One-way ANOVA with Tukey’s post hoc test. **h** αSyn A53T mice and wildtype littermates were i.c.v-injected with AAV-control or AAV-ClpP at the age of 6 months. Mouse behavior was assessed once per month after AAV injection. A locomotion activity chamber was used to monitor 24 h of general motility of animals at the indicated age (*n* = 12 mice/group). Shown is vertical activity. **p < 0.01 vs. wildtype mice injected with AAV-control; #*p* < 0.05 vs. αSyn A53T mice injected with AAV-control. ANOVA with Tukey’s post hoc test. All data are mean ± SEM. **i** A scheme of summary

Next, we examined whether compensating for the loss of ClpP would attenuate αSyn-associated neuropathology in vivo. To drive the expression of ClpP in vivo, we adopted an adeno-associated virus (AAV)-mediated gene delivery strategy by which we virally overexpressed ClpP in the SN of 6-month-old αSyn A53T or age-matched Wt mice (Fig. 7a). The AAV containing ClpP is under the control of the hSynapsin promoter (AAV5-hSyn-eGFP-ClpP), and thus selectively expressed in neurons (Fig. 7a). Two months after stereotaxical injection in both SNs, we observed that eGFP-labeled AAVs containing ClpP was successfully delivered; enhanced ClpP expression as evidenced by the presence of eGFP (green), was observed in more than 70% of TH^+^-marked DA neurons (red) (Fig. 7a). Compared to the mice injected with AAV-eGFP control, western blot analysis showed a strong upregulation of ClpP in nigral tissue extracts of AAV5-ClpP-injected mice 4 months after injection (Fig. 7b). Moreover, overexpression of AAV-ClpP induced an up-regulation of endogenous ClpP in the nigra of both Wt and αSyn-A53T mice (Fig. 7b).

Consistent with the results in our cell culture, the protein level of SOD2 significantly decreased in the midbrain containing the SN of αSyn A53T mice injected with AAV-eGFP-control (Fig. 7b), which was rescued in AAV-ClpP-injected αSyn A53T mice (Fig. 7b). We observed an elevated amount of TPE-MI-fluorescence-labeled mitochondrial unfolded proteins and an increased level of oxidized mitochondrial proteins in the nigral tissue of αSyn A53T mice injected with AAV-control (Fig. 7c, d), suggestive of misfolded/unfolded protein accumulation. By contrast, viral expression of AAV-ClpP attenuated these abnormalities (Fig. 7c, d). A great increase in the immunodensity of αSyn-pS129 in the SN of αSyn-A53T mice injected with AAV-control was observed at the age of 10 months, and viral expression of AAV-ClpP starting from the age of 6 months reduced the levels of αSyn-pS129 (Fig. 7e, f). Consistently, western blot analysis showed that AAV-ClpP expression abolished increased level of αSyn-pS129 in the midbrain containing the SN of αSyn A53T mice (Fig. 7g). In contrast, AAV-ClpP expression did not influence αSyn total protein level nor the distribution of αSyn in the soluble and insoluble fractions of mouse brains (Suppl Fig. 6g). The mechanism by which overexpression of ClpP reduces pathological αSyn S129 phosphorylation remains to be investigated.

In parallel, we examined the behavioral activity of the animals in the presence or absence of AAV-ClpP. Consistent with previous studies [68, 84], αSyn A53T mice exhibit age-dependent vertical hyperactivity measured by open-field locomotion chambers starting from the age of 6 months. We found that AAV-ClpP expression significantly reduced vertical hyperactivity of αSyn A53T mice 1 month after the AAV injection, and the protection lasted 4 months until these mice were killed at the age of 10 months (Fig. 7h). Note that expression of AAV-ClpP in Wt mouse brains did not alter animal behavioral status (Fig. 7h), suggesting a less toxic effect during the viral expression period. Collectively, these results demonstrate that up-regulation of ClpP in the SN reduces mitochondrial oxidative damage and attenuates αSyn-associated pathology and behavioral phenotype in αSyn-A53T mice.

## Discussion

In this study, we have identified, for the first time, an important role of mitochondrial matrix protease ClpP in αSyn-associated neuropathology (Fig. 7i). First, αSyn induces a selective loss of ClpP in DA neurons in both αSyn A53T transgenic mice and PD patients, which results in an overload of mitochondrial misfolded/unfolded proteins and enhanced oxidative damage. Second, αSyn and A53T mutant interact with ClpP, which impairs ClpP proteolytic activity and promotes ClpP insolubility and aggregation. Third, compensation for the loss of ClpP in both neurons derived from PD patient and the SN of αSyn A53T transgenic mice reduces mitochondrial oxidative damage and αSyn-associated neuropathology. Thus, our findings reveal a novel mechanism by which αSyn induces mitochondrial damage to proceed PD-associated neuropathology.

ClpP has been widely studied in prokaryotic cells as an important component of the proteasome-like complex ClpXP/ClpXA [31]. The primary physiological role of ClpP is to maintain cellular homeostasis by degradation of short-lived proteins as well as misfolded or damaged proteins, including those involved in regulating stress responses and virulence factor production [5]. Therefore, ClpP plays an essential role in the virulence of pathogenic bacteria during host infection [89]. In *C. elegans*, ClpP was previously proposed as a component involved in UPR^mt^, maintaining proteostasis in the mitochondrial matrix [38]. In dopaminergic SH-SY5Y cells, upon ClpP silencing, we observed extensive mitochondrial defects, consistent with previous reports in mitochondria isolated from heart and skeletal muscle tissue from ClpP null mice [29] and in C2C12 cells with ClpP knockdown [22]. A decline in mitochondrial inner and intermembrane proteases, such as PARL, YME1L1, OPA1 and HtrA2, is associated with mitochondrial respiratory defects, oxidative damage, and neurodegeneration [22, 52, 69]. Our findings, thus, provide another line of evidence to support the notion that mitochondrial proteases, located in different sub-compartments of mitochondria, carry out critical steps of mitochondrial protein turnover, defects of which contribute to the development of neurodegenerative diseases [69].

ClpP deficiency causes Perrault syndrome characterized by sensorineural hearing loss and premature ovarian failure in humans [25, 42]. ClpP knockout mice exhibit growth retardation, mtDNA accumulation, inflammatory response and infertility, recapitulating the pathology of Perrault syndrome in humans [29]. Interestingly, whole-body deletion of ClpP in mice was recently reported to protect against diet-induced obesity and insulin resistance [8]. However, ClpP ablation in brown adipocytes leads to a decline in brown adipocytes function, leaving mice unable to cope with cold-induced stress because of non-functional adaptive thermogenesis [7]. Conditional knockout of ClpP in heart tissue in DARS2-deficient mice attenuates mitochondrial cardiomyopathy [75]. In our study, we showed that ClpP was pathologically decreased by αSyn in DA neurons of mice and patients due to the interaction and co-aggregation with αSyn, and that compensation for the loss of ClpP in the SN of αSyn A53T mice reduces αSyn-related neuropathology and behavioral deficits. Thus, it is likely that ClpP functions in a tissue- or organ-dependent manner in response to stresses. Because of technical limitations, the neuronal cultures derived from iPS cell model by our differentiation protocol contain 20–30% TH^+^ neurons ([80] and Suppl Fig. 2). In the immunostaining assay, we stained cells with anti-TH to mark DA neurons, which ensures our ability to make observations in these neurons. However, at times with western blot analysis, it is difficult to discern the extent to which ClpP is decreased in TH^+^ and non-TH^+^ cells. A future comparison of the differences in ClpP protein level, mitochondrial activities, and neuronal abnormality between DA and non-DA neurons derived from PD patient iPS cells may further distinguish the cell type specificity of ClpP deficiency-mediated neuronal toxicity in the context of PD patient phenotypes.

The higher toxicity of αSyn A53T in different aspects of mitochondria has been widely reported in vitro and in vivo [17, 23, 65]. The nature of a greater mitochondrial translocation and aggregation of αSyn A53T than αSyn WT has also been recognized by many independent studies in both exogenous and endogenous contexts [17, 23, 49, 50, 76]. It is possible that the extent of ClpP decreasing is in direct proportion to the level of αSyn WT and A53T accumulated in the mitochondria. However, in our in vitro tube assay, we found that αSyn A53T was more toxic on the peptidase activity of ClpP and prone to co-aggregate with ClpP, compared to αSyn WT. These results suggest another possibility that, relative to αSyn WT, the higher toxicity of αSyn A53T on ClpP may come from its intrinsic nature (single point mutation). Combining with these facts, we speculate that more severe suppression of ClpP by αSyn A53T could be a comprehensive result from both αSyn A53T intrinsic toxicity and its mitochondrial accumulation.

Although αSyn A53T transgene is highly expressed in all brain regions [47], aberrant αSyn accumulations are limited to a subset of neuronal populations in the midbrain, cerebellum and brainstem [20, 47, 71]. In the SN of αSyn A53T mice exhibiting αSyn aggregates, we clearly observed localization of ClpP in TH^+^ neuron (Fig. 2). Moreover, we found a selective decrease of ClpP in these DA neurons (Fig. 2). In contrast, no change in the protein level of ClpP in other brain regions (such as cortex, brainstem and striatum) was observed in the A53T mice. This region vulnerability of ClpP was validated in PD patient postmortem brains. αSyn is enriched in the mitochondria of SN compared to other brain regions, predisposing the neurons to degeneration [81, 82]. αSyn A53T mutant is more preferentially accumulated in the mitochondria of PD vulnerable brain regions [23, 81]. Such brain region and mitochondrial enrichment of pathological αSyn may explain the selective decrease of ClpP in vivo. Future investigation on the brain region susceptibility of ClpP to αSyn toxicity might shed new light on the mechanism of DA neuron degeneration in PD. ClpP complexes with its AAA+ chaperone ClpX to function as a proteasome-like protein turnover machinery in the mitochondrial matrix. However, neither αSyn WT nor A53T mutant affects ClpX in culture and in mice, nor do they interact with ClpX. Instead, αSyn and A53T mutant interacted with ClpP, which in turn suppressed ClpP proteolytic activity. Thus, direct interaction with ClpP and inhibition of ClpP enzymatic activity by αSyn may ultimately result in a selective decrease of ClpP protein level in the αSyn-enriched brain region.

Certain species of αSyn have been reported to impair mitochondrial protein import by directly interacting with Tom20 to prevent Tom20–Tom22 interaction [24]. However, in our study, the fact that the mitochondrial matrix proteins LonP and ClpX were unchanged in αSyn models in vitro and in vivo seems to exclude the possibility that decreasing ClpP by αSyn is due to the impairment of mitochondrial import. In future studies, it will be interesting to distinguish the population of mitochondrial proteins that is influenced indirectly by αSyn via the mitochondrial import impairment or directly by mitochondrial accumulated αSyn. Our discovery of ClpP decreasing by αSyn may add to the list of proposed mechanisms of αSyn-induced mitochondrial dysfunction.

There is a great deal of disagreement over which rodent model is appropriate to use as a platform for the study of PD in the field. Various toxic and transgenic models of PD are currently available, all with significant advantages and disadvantages, and few of the current PD animal models fulfill all of the key features of PD [9, 33, 48]. Thus, until the ‘perfect’ model is developed, using one model or another will depend on the specific needs. Owing to the selective loss of ClpP in the SN of αSyn A53T mice and PD patients, we focused on the SN and stereotaxically injected AAV-ClpP in this specific brain region. Utilization of neuronal cells derived from PD patient iPS cells carrying αSyn A53T mutant compensates for the limitation of the αSyn A53T transgenic mice in which DA neuronal degeneration is lack, a caveat in fact existing in most of current PD transgenic mouse models [9, 48]. More recently, Nuber et al. reported a new αSyn transgenic mouse model that expresses tetramer-lowering “3K” αSyn mutation. The mouse line recapitulates many pathological phenotypes seen in human PD, particularly dopaminergic neuronal degeneration [62]. The predominant post-translational modification of 3K αSyn mutation is extensive Ser129 phosphorylation [62]. Given that expression of AAV-ClpP abolished αSyn S129 phosphorylation with a minor effect on αSyn protein level, it will be of interest to determine whether expression of AAV-ClpP could stabilize αSyn tetramer, which in turn would reduce DA neuronal degeneration in that mouse line.

In the current study, we showed that, though to a lesser extent than αSyn A53T, αSyn WT can be translocated into mitochondria, and affects the peptidase activity of ClpP and co-aggregates with ClpP in vitro. The vast majority of sporadic PD cases show a 2-4fold overexpression of WT αSyn [16]. One can image that in the context of sporadic PD, 2-4fold overexpression of αSyn WT would impair the function of ClpP, which could induce mitochondrial dysfunction and neuronal damage. Indeed, our results showing a great decrease in the protein level and immunodensity of ClpP in the SN of sporadic PD patients, support such possibility. Therefore, we propose that ClpP decreasing might be a common event across sporadic and familial PD manifesting αSyn pathology. Notably, our results demonstrate that enhancement of ClpP in both the SN of PD mice and DA neurons of PD patient reduced αSyn-associated pathology. Thus, our findings should stimulate the development of ClpP modulators as potential disease-modifying therapeutic agents in PD and other synucleinopathies.

## Electronic supplementary material

Below is the link to the electronic supplementary material.

Supplementary material 1 (PDF 6581 kb)

## Data Availability

The datasets generated during and/or analyzed during the current study are available from the corresponding author on reasonable request.
