# Supplementary material for: Alpha-synuclein suppresses mitochondrial protease ClpP to trigger mitochondrial oxidative damage and neurotoxicity
Source: Acta Neuropathol. 2019 Mar 15;137(6):939–60. doi: 10.1007/s00401-019-01993-2 (PMC6531426; doi:10.1007/s00401-019-01993-2)

Supplementary Information

Alpha-Synuclein suppresses mitochondrial protease ClpP to trigger mitochondrial oxidative damage and neurotoxicity

Di Hu[1], Xiaoyan Sun[1], Xudong Liao[2], Xinwen Zhang[1,3], Sara Zarabi[4], Aaron Schimmer[4], Yuning Hong[5], Christopher Ford[6], Yu Luo[7] and Xin Qi [1*]

[1]Department of Physiology & Biophysics, Case Western Reserve University School of Medicine, Cleveland, OH 44106, USA; [2]Case Cardiovascular Research Institute, Case Western Reserve University School of Medicine, Harrington Heart and Vascular Institute, University Hospitals Cleveland Medical Center; [3]Center of Implant Dentistry, School of Stomatology, China Medical University, Shenyang 110002, China; [4]Princess Margaret Cancer Centre, Toronto, ON M5G 2M9, Canada; [5]Department of Chemistry and Physics, La Trobe Institute for Molecular Science, La Trobe University, Melbourne VIC 3083; [6]Department of Pharmacology, University of Colorado, Denver; [7]Department of Molecular Genetics, University of Cincinnati, OH, USA.

***Corresponding author:**

Xin Qi PhD, Department of Physiology and Biophysics, Case Western Reserve University School of Medicine, 10900 Euclid Ave, E516, Cleveland, Ohio, 44106-4970, USA. Tel: 216-368-4459; Fax: 216-368-5586; E-mail: xxq38@case.edu

Supplemental Figure 1: (a) Mitochondrial fractions were isolated from SH-SY5Y cells stably expressing GFP control, GFP-αSyn WT or GFP-αSyn A53T mutant, followed by fractionating into Triton-X100-soluble and -insoluble fractions. αSyn level was examined by western blot analysis. VDAC was used as a mitochondrial marker in soluble fractions. Shown blots are representative of 2 independent experiments. (b) Mitochondrial, ER and

cytosolic fractions were isolated from SH-SY5Y cells stably expressing GFP control, GFP-αSyn WT, or GFP-αSyn A53T mutant. Purity of mitochondrial fractions was examined by Western blot analysis with the indicated antibodies. VDAC: a mitochondrial marker; WFS1: an ER marker; Enolase: a cytosolic marker. Shown blots are representative of 2 independent experiments. (c) Mitochondria were isolated from HEK293 cells transfected with Myc, Myc-αSyn WT, and Myc-αSyn A53T, followed by protease K digestion. Mitochondrial fractions after protease K digestion were subjected to western blot analysis with the indicated antibodies. MFN1: mitofusin 1, a mitochondrial outer membrane protein; ClpX: a mitochondrial matrix protein. Shown blots are representative of 2 independent experiments. (d) The mRNA level of ClpP was examined by qPCR in SH-SY5Y cells stably expressing GFP control, GFP-αSyn WT or GFP-αSyn A53T mutant. Data are mean ± SE of three independent experiments. (e) Neuronal cells were differentiated from iPS cells of PD patient carrying αSyn A53T mutant and isogenic corrected control for 40 days. Total cell lysates from the mixture of neuronal cells were harvested and subjected to western blot analysis with the indicated antibodies. Histogram: quantitative protein density of western blots shown in Fig. 1c from each of independent differentiation batch. Data are mean ± SE.

Supplement Figure 2: Neuronal cells were differentiated from iPS cells of PD patient carrying αSyn A53T mutant or isogenic corrected control. Using the protocol described in the Method, the efficiency of neuronal differentiation was 60-70% and the yield of TH dopaminergic neurons was 20-30 %. (a) Cells were stained with anti-TH (green), anti-beta-Tubulin III (Tuj1, red), and DAPI (blue). Scale bar: 10 µm. The length of neurites in TH+/Tuj1+ cells was analyzed and quantitated. At least 20 neurons/group were analyzed. Data are mean ± SE. Student *t*-test. (b) Cells were stained with anti-MAP2 (Green), anti-TH (red) and DAPI (blue). Scale bar: 10 µm. The length of neurites in MAP2+/TH+ cells was quantitated. At least 20 neurons/group were analyzed. Data are mean ± SE. Student *t*-test. (c) Cells were stained with anti-αSyn pS129 (red), anti-TH (green) and DAPI (blue). Scale bar: 10 µm. The image are representative of 3 independent experiments. (d) Total cell lysates of iPS cells from PD patients carrying αSyn A53T mutant or its isogenic corrected control (ctl) were harvested and subjected to western blot analysis with the indicated antibodies. Shown blots are representative of 3 independent experiments. Data are mean ± SEM of 3 independent experiments. Student *t*-test. (e) Histogram: quantitation of ClpP mRNA

level in neurons derived from iPS cells of PD patients carrying A53T αSyn mutant or isogenic corrected control. Data are mean ± SEM of 3 independent experiments. Student *t*-test.

Supplemental Figure 3: (a) Cortex, brainstem and striatum were harvested from wildtype littermates (Wt) and αSyn A53T mice at the age of 8 months. Western blot analysis was performed using the indicated antibodies. Actin was used as a loading control. n=4 mice/group. (b) Mitochondrial fractions were isolated from the midbrains containing the SN of wildtype (Wt) and αSyn A53T mice at the age of 8 months. Mitochondrial fractions were subjected to western blot analysis with anti-αSyn antibodies. VDAC was used as a mitochondrial loading control. Shown blots are representative of 3 independent experiments. (c) The mRNA level of ClpP in Wt and A53T mouse SN (10 months old) was examined by qPCR. The data are mean ± SEM. n=3 mice/group. Student *t*-test. (d) Total protein lysates were harvested from the midbrains containing the SN of wildtype (Wt) and αSyn A53T mice at the age of 8 months. The level of tyrosine hydroxylase (TH) was examined by western blot analysis. Actin was used as a loading control. Data are mean ± SEM. n=3 mice/group. There was comparable level of TH between wildtype and αSyn-A53T mice at this age. (e) Brain sections of wildtype (Wt) and αSyn A53T mice were stained with anti-ClpP (green) and anti-NeuN (red, a neuronal marker) antibodies. There was no noticeable difference on ClpP immunodensity in the cortex between Wt and αSyn A53T mice. Nuclei were stained with DAPI (blue). Scale bar: 10 µm. (g) The SN sections of PD patients (ID: 4879, 4986 and 5203) and normal subjects (ID: 625, 2803 and 5103) were stained by anti-αSyn antibodies. PD patients showed αSyn accumulation in the SN. Scale bar: 10 µm. The information of PD patients and normal subjects was listed in (f). (h) Total protein lysates were obtained from the frozen cortex of 6 PD patients and 6 control subjects. Western blot analysis was performed with the indicated antibodies. Histogram: quantitation of ClpP protein density to actin. The information of frozen brain samples of PD patients and normal subjects was listed in (i).

Supplemental Figure 4: (a) mitochondrial respiratory activity in SH-SY5Y cells stably expressing GFP-control, GFP-αSyn WT or GFP-αSyn A53T was measured by seahorse analyzer. Maximal OCR, basal OCR and ATP content were calculated and shown in histograms. Data are mean ± SE of 3 independent experiments. One-way ANOVA with Tukey's *post-hoc* test (b) Mitochondrial protein oxidation in SH-SY5Y cells stably expressing

GFP-control, GFP-αSyn WT or GFP-αSyn A53T was determined. Neg Ctl: negative control. Shown blots are representative of 3 independent experiments. Histogram shows the intensity measurement of oxidized proteins in contrast to mitochondrial loading control VDAC. One-way ANOVA with Tukey's *post-hoc* test. (c) iPS cell colony of αSyn A53T PD patient and isogenic corrected control were stained with MitoSOX probe that indicates mitochondrial superoxide production. Left: representative images. Scale bar: 10 μm. Right: quantitation of fluorescence density of MitoSOX red/cell. Data are mean ± SE of 3 independent experiments. Student *t*-test. (d) Mitochondrial respiratory activity in the iPS cell colony was measured using seahorse analyzer. Maximal OCR, basal OCR and ATP content were calculated and shown in histograms. Data are mean ± SE of 3 independent experiments. Student *t*-test. (e) Neuronal cells were differentiated from iPS cells of PD patient carrying αSyn A53T mutant or isogenic corrected control. 40 days after neuronal differentiation, cells were stained with mitoSOX red followed by staining cells with anti-TH antibody and DAPI, and the mitoSOX fluorescence density in TH+ cells was quantitated to indicate mitochondrial superoxide production. Scale bar: 10 μm. At least 50 neurons/group were counted. Student *t*-test. (f) Mitochondrial respiratory activity in the mixed neuronal cells was measured using seahorse analyzer. Maximal OCR, basal OCR and ATP content were calculated and shown in histograms. Data are mean ± SE from 3 differentiation batches with duplicated samples in each of differentiation batch. Student *t*-test. (g) Cell death in mixed neuronal cells derived from iPS cells of αSyn A53T patient and isogenic corrected control was determined by measuring the LDH release into medium. Data are mean ± SE from 3 differentiation batches. Student *t*-test.

Supplemental Figure 5: (a) Total cell lysates of iPS cells from PD patients carrying αSyn A53T mutant or its isogenic corrected control were harvested, and subjected to western blot analysis with the indicated antibodies. Shown blots are representative of 3 independent experiments. Data are mean ± SEM of 3 independent experiments. Student *t*-test. (b) HEK293 cells were transfected with random-dosed control (Ctl) or ClpP siRNA for 3 days. Total protein lysates were subjected to western blot analysis with the indicated antibodies. Data show the representative blot of 3 independent experiments. (c) A linear correlation of the relative density of ClpP and SOD2 in contrast to Actin was shown.

Supplemental Figure 6: (a) Digitonin-soluble and -insoluble fractions were isolated from SH-SY5Y cells stably expressing GFP control vector (GFP), GFP-αSyn WT (WT), or GFP-αSyn A53T (A53T), and were then subjected to western blot analysis with the indicated antibodies. Quantitative analysis of protein expression levels was performed by intensity measurement of ClpP in contrast to β-actin. Data are mean ± SE of 3 independent experiments. One-way ANOVA with Tukey's *post-hoc* test. (b) Digitonin-soluble and -insoluble fractions were isolated from the midbrains of αSyn A53T and wildtype mice at the indicated ages, and were then subjected to western blot analysis with the indicated antibodies. n=4 mice/group. Data are mean ± SE. One-way ANOVA with Tukey's *post-hoc* test. (c) HEK293 cells were expressed with GFP control, GFP-αSyn WT or A53T mutant for 2 days. Total lysates of cells were immunoprecipitated with anti-GFP antibodies, followed by immunoblotting with anti-ClpX and anti-GFP antibodies. Data are representative of 2 independent experiments. (d) HEK293 cells were expressed with Myc control, Myc-αSyn WT or A53T mutant for 2 days. Total lysates of cells were immunoprecipitated with anti-Myc antibodies, followed by immunoblotting with anti-LonP and anti-Myc antibodies. Data are representative of 2 independent experiments. (e) Recombinant ClpP proteins with different concentration (1, 5, 10 μM) were incubated with ac-WLA-AMC (50 μM), and the relative fluorescence unit (RFU) was determined for 30 min. Data are representative of 2 independent experiments. (f) Left: SH-SY5Y cells were transfected with control siRNA or ClpP siRNA for 3 days. Right: SH-SY5Y cells were transfected with Myc vector, Myc-ClpP-WT or Myc-ClpP-S153A mutant for 2 days. Total cell lysates were harvested and subjected to western blot analysis with the indicated antibodies. Histogram: quantitative protein density to actin. Data are mean ± SE of 3 independent experiments. Left: Student *t*-test, Right: One-way ANOVA with Tukey's *post-hoc* test. (g) The midbrains containing the substantia nigra (SN) of wildtype (Wt) and αSyn A53T (A53T) mice were harvested 4 months after injection of AAV control vector or AAV-GFP-ClpP. Digitonin-soluble and -insoluble fractions were isolated from the midbrain of αSyn A53T and wildtype mice at the indicated ages, and were then subjected to western blot analysis with the indicated antibodies. Data are representative image of 4 independent experiments. n=4 mice/group.

Supplementary Figure 1

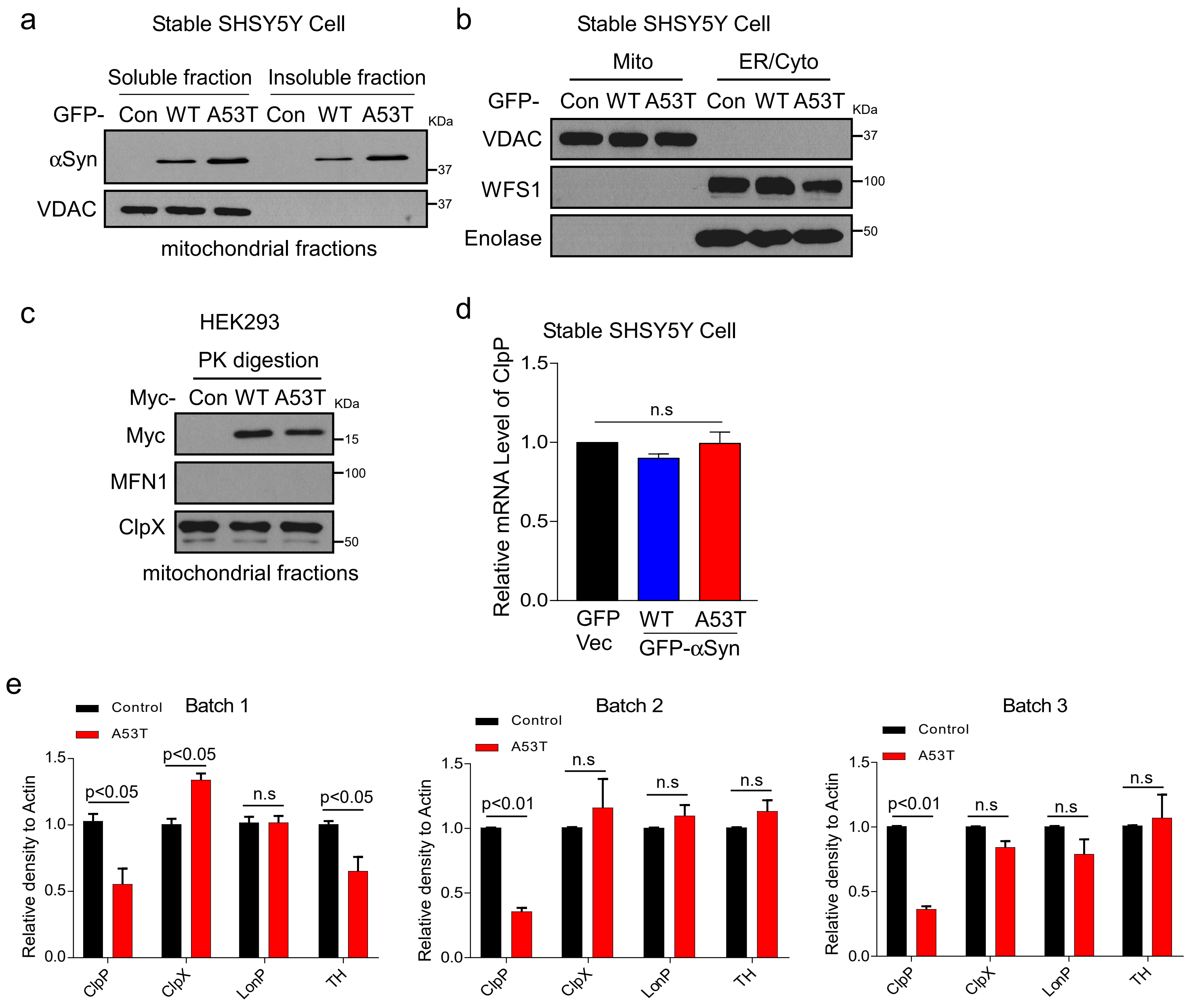

Supplementary Figure 2

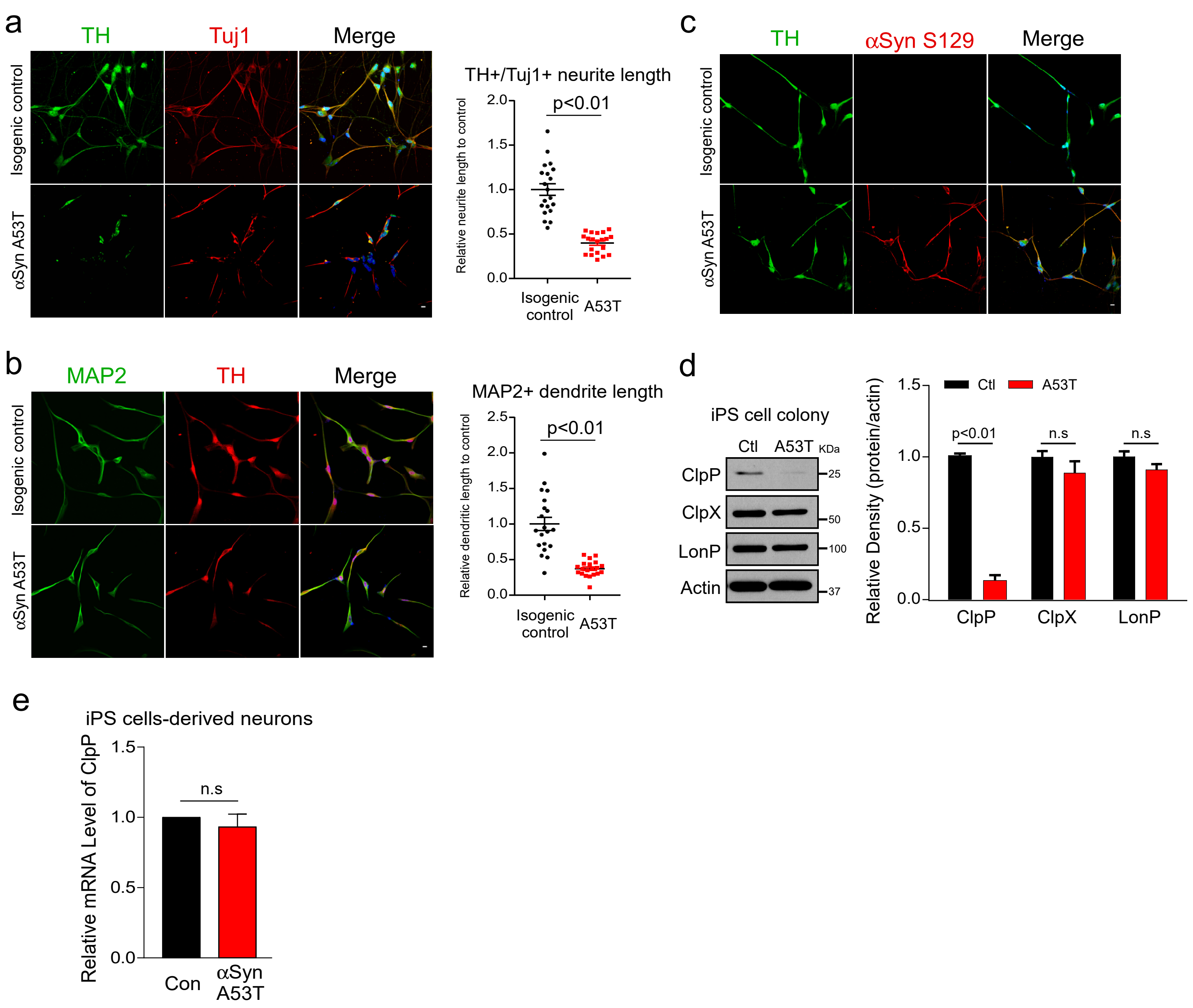

Supplementary Figure 3

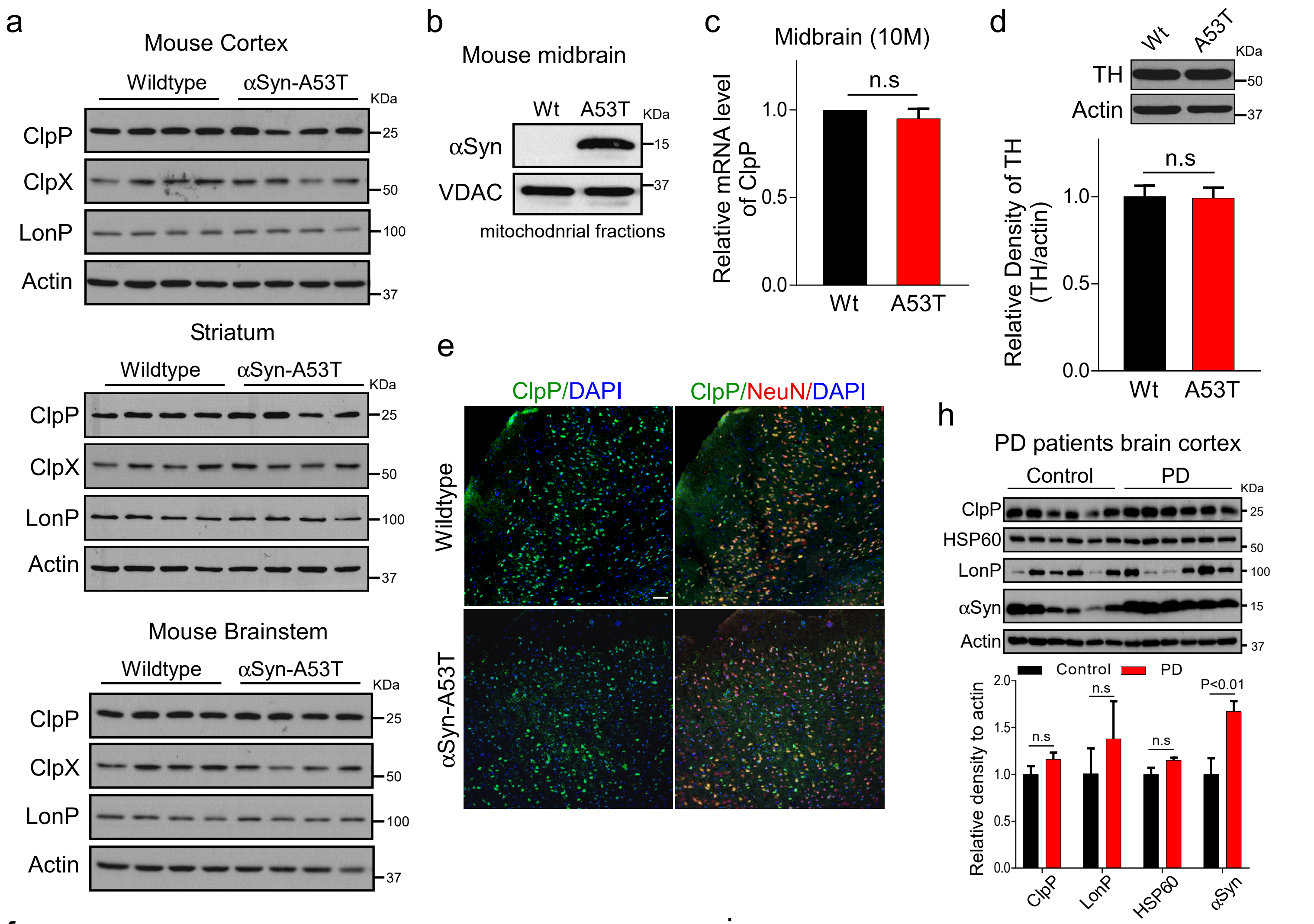

f

PD patients and normal subjects postmortem brain samples (parafin embeded)					
ID	Age	Gender	phenotypes	structure	Race
625	87	F	normal	Substantia Nigra	Caucasian
2803	92	M	normal	Substantia Nigra	Caucasian
5103	78	M	normal	Substantia Nigra	Caucasian
5203	89	M	PD with lewy body disease	Substantia Nigra	Caucasian
4879	75	M	PD with lewy body disease	Substantia Nigra	Caucasian
4986	86	F	PD with lewy body disease	Substantia Nigra	Caucasian

i

PD patients and normal subjects postmortem brain				
ID	Age	Gender	phenotypes	Race
4593	76	M	Unaffected Control	Unknown
4631	59	M	Unaffected Control	Unknown
5190	68	M	Unaffected Control	Unknown
HCTYP_18_02_SN	75	M	Unaffected Control	Caucasian
HCTYM_18_06_SN	74	M	Unaffected Control	Caucasian
HCTZT_18_08_SN	76	M	Unaffected Control	Caucasian
848	74	M	PD	Unknown
4349	61	M	PD	Unknown
5198	71	M	PD	Unknown
HBEO_18_10_SN	74	M	PD	Caucasian
HBEW_18_12_SN	79	M	PD	Caucasian
6248	71	M	PD	Caucasian

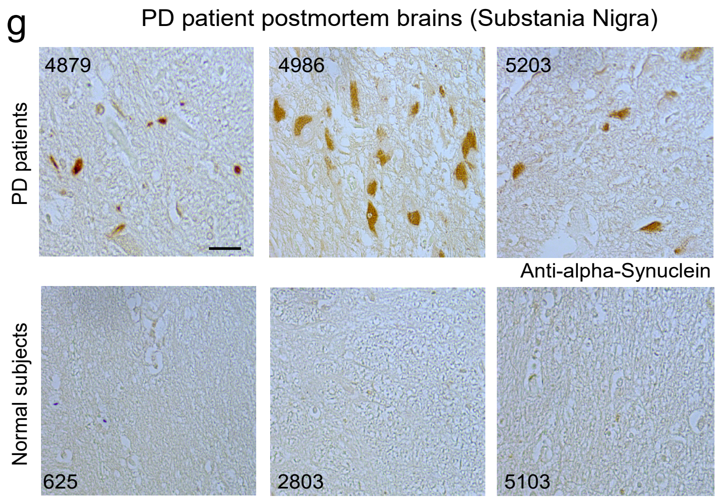

Supplementary Figure 4

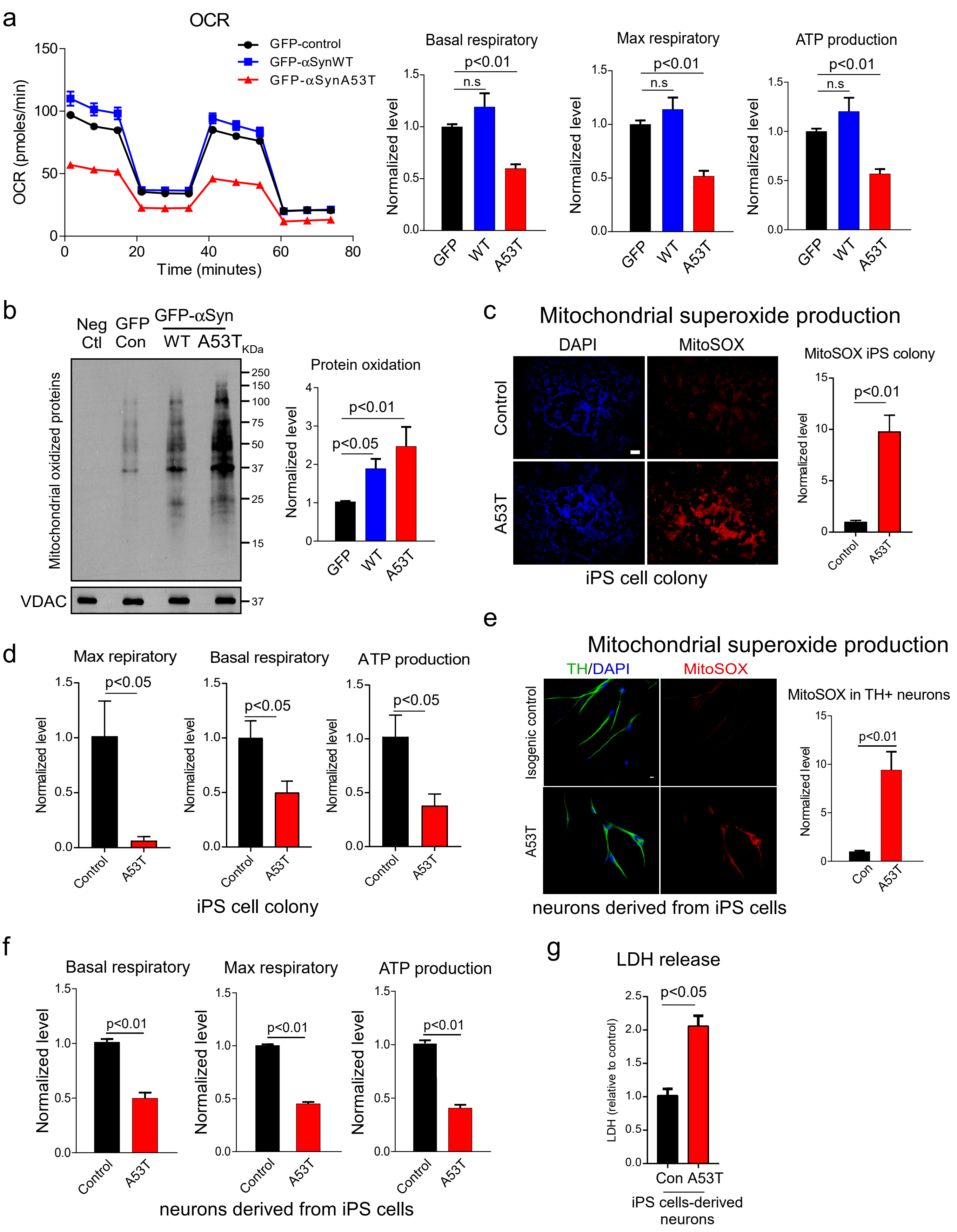

Supplementary Figure 5

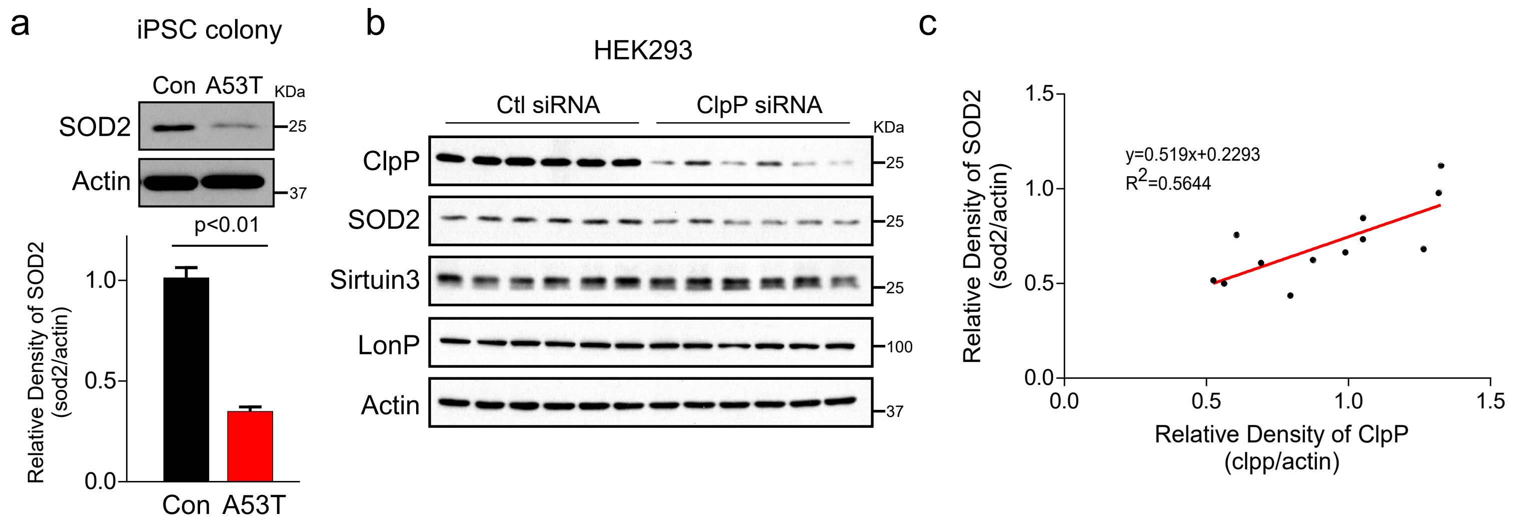

Supplementary Figure 6

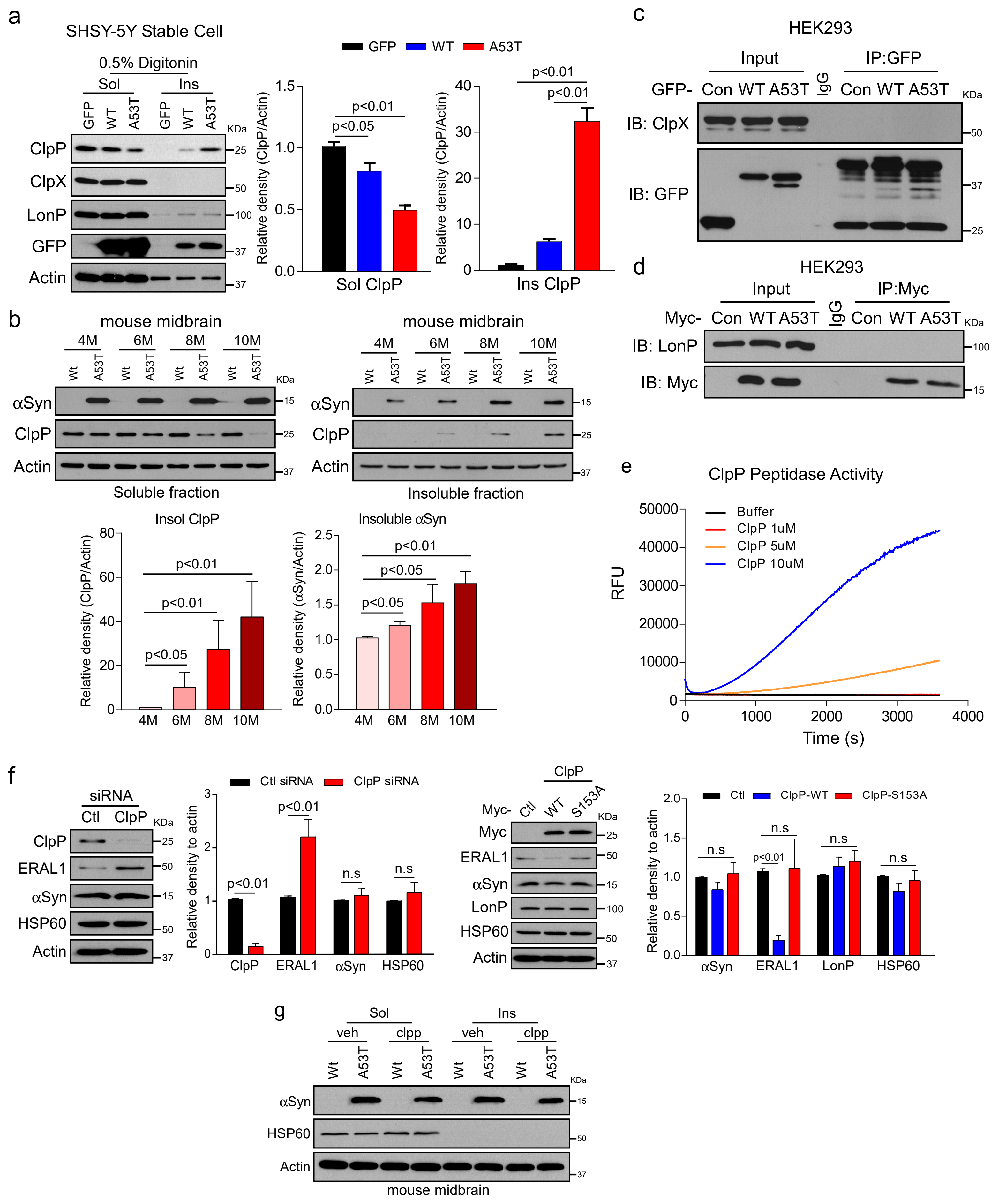

Supplement: Supplementary file 1 — Supplementary material 1 (PDF 6581 kb) [file 401_2019_1993_MOESM1_ESM.pdf]
